# Large-Scale Prediction of Drug-Target Interaction: a Data-Centric Review

**DOI:** 10.1208/s12248-017-0092-6

**Published:** 2017-06-02

**Authors:** Tiejun Cheng, Ming Hao, Takako Takeda, Stephen H. Bryant, Yanli Wang

**Affiliations:** 1 National Center for Biotechnology Information, National Library of Medicine, National Institutes of Health, Bethesda, MD 20894, USA.

**Keywords:** compound-protein interactions, drug repositioning, drug-target interactions, public databases

## Abstract

The prediction of drug-target interactions (DTIs) is of extraordinary significance to modern drug discovery in terms of suggesting new drug candidates and repositioning old drugs. Despite technological advances, large-scale experimental determination of DTIs is still expensive and laborious. Effective and low-cost computational alternatives remain in strong need. Meanwhile, open-access resources have been rapidly growing with massive amount of bioactivity data becoming available, creating unprecedented opportunities for the development of novel in silico models for large-scale DTI prediction. In this work, we review the state-of-the-art computational approaches for identifying DTIs from a data-centric perspective: what the underlying data are and how they are utilized in each study. We also summarize popular public data resources and online tools for DTI prediction. It is found that various types of data were employed including properties of chemical structures, drug therapeutic effects and side effects, drug-target binding, drug-drug interactions, bioactivity data of drug molecules across multiple biological targets, and drug-induced gene expressions. More often, the heterogeneous data were integrated to offer better performance. However, challenges remain such as handling data imbalance, incorporating negative samples and quantitative bioactivity data, as well as maintaining cross-links among different data sources, which are essential for large-scale and automated information integration.

## INTRODUCTION

**Fig. 1.**
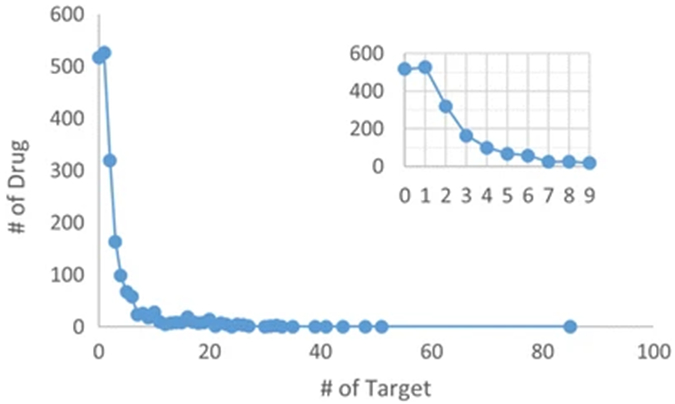
Distribution of drug as a function of the number of target. The statistics is based on about 2000 approved small-molecule drugs and their associated targets in DrugBank. The analysis shows that while the majority of drugs have one or a few targets with three targets per drug on the average, some drugs are “promiscuous” and have multiple targets. One extreme drug, *Flavin adenine dinucleotide* (DrugBank ID: DB03147), has 85 targets according to DrugBank

Human health nowadays has been considerably improved through medical interventions. However, many diseases remain poorly treated while new ones are emerging. Some complex diseases such as cancer and neurodegenerative disorders still lack efficient therapies. Moreover, drugs are not available for many rare and neglected diseases due to little interest from pharmaceutical companies. Unfortunately, drug development is costly and lengthy while existing drugs may become less effective due to drug resistance. Despite enormous investments and advances, the number of approved drugs has not increased proportionally. The therapeutic dogma entered on “one drug-one target-one disease,” which has prevailed in the industry over the years, is now being challenged. It is becoming evident that drugs can interact with other targets in addition to that aimed primarily. According to DrugBank, one drug can have three targets on average (Fig. 1). Such off-target interactions often lead to adverse side effects or toxicity, but they also open the door to drug repositioning (1), an appealing drug discovery strategy that potentially is cheaper, faster, and less risky that can result in identifying new indications for old drugs. Furthermore, growing evidence shows that several drugs exert their effects through interactions with multiple targets in a complex system (2). These findings have promoted a shift from single to multi-target paradigm in drug discovery (3), which is especially helpful in treating complex diseases like cancer or central nervous system disorders, where the modulation of one single protein is often insufficient to accomplish a desired therapeutic effect. Consequently, the identification of potential drug-target interactions (DTIs) is of critical importance in many applications, such as suggesting new uses for existing drugs, identifying drug candidates for known therapeutic targets, and understanding the pharmacological actions for those approved drugs without known targets (Fig. 1).

Given the high cost of wet-lab experiments, in silico methods have been developed and proven successful to explore the potential DTIs (4). Conventional approaches fall roughly into two categories: ligand-based and structure-based, e.g., quantitative structure activity relationship (QSAR) (5), pharmacophore modeling (6), or molecular docking (7). Many recent studies have focused on chemogenomic approaches taking advantage of the rapid growth of the large-scale chemical biology data in the public domain, such as those provided by PubChem BioAssay (8-10) and ChEMBL (11). Depending on how the DTI prediction problem is generalized, there can be various solving strategies. For example, the interaction space of drugs and targets can be represented as a bipartite graph with nodes being drugs and targets and edges being their interactions. The task of predicting novel DTIs is equivalent to finding “missing” links in the graph. Accordingly, graph-based and network-based analysis can be applied. Similarly, the bipartite graph can be transformed into an association matrix and “hidden” associations can be inferred through methods like matrix factorization. In machine learning methods, drugs and targets are expressed as features, with their interactions being denoted as class labels. A potential DTI can be determined by the predicted class label. There are other possible solutions, and some recent reviews focused on the technical aspect of mathematical or statistical methods (12-17). Regardless, prior knowledge of drugs, targets, and their interactions is required for all in silico method development. The growth of publicly available chemical biology data is expected to continue at an accelerating rate with high-throughput screening (HTS) becoming affordable at universities and academic institutions, and with further policy implementation of mandatory data sharing (https://grants.nih.gov/grants/policy/data_sharing/data_sharing_guidance.htm), which provides huge opportunities for DTI modeling by incorporating various information resources for drugs, compounds, and knowledge about their therapeutic targets.

In this work, we provide a data-centric review on applications/approaches in the past 5 years for predicting DTIs with a primary focus on data, i.e., data categories and utilities in each study. We first summarize a number of commonly used public databases for predicting DTIs. Then we describe selected approaches according to the underlying data type, mostly from the drug side, followed by a list of online tools. Finally, we highlight some recent trends in DTI prediction studies as well as challenges from the data perspective. We hope our review will be helpful towards designing more accurate and robust approaches through better consideration over the nature of the data, which will eventually facilitate the process of drug discovery and repositioning.

## DATA RESOURCES

**Fig. 2.**
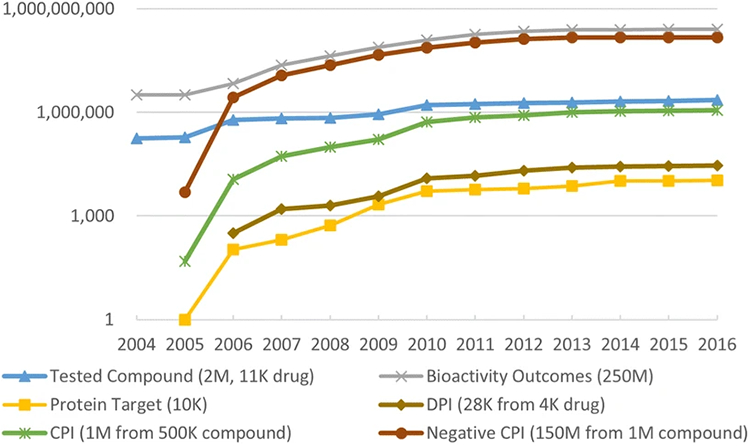
The growth of biological data in PubChem BioAssay including biologically tested compounds, bioactivity outcomes, protein targets, drug-protein interactions (DPIs), compound-protein interactions (CPIs), negative compound-protein interactions (CPIs). The number in parenthesis is the total count of each data category. DPI and CPI are counted based on the confirmatory and literature-based assays

As a premise for predicting DTIs, it is necessary to collect as much data as possible on drugs, targets, and their interactions. With the advent of new technologies and open data initiatives, the past decade has witnessed an exponential growth of chemical biology data available in the public databases (18-40). As an example, PubChem currently contains over two million compounds tested in biochemical or cell-based assays generating 250 million bioactivity outcomes (Fig. 2). Over a half million of those compounds, including 4000 drugs, were biologically tested against 11,000 protein targets, and more than one million compound-protein interactions (CPIs) were reported. The numerous biological data, with dramatically increased volume and diversity, have created unprecedented opportunities for developing novel algorithms and online tools for DTI prediction. In this review, we focus on a subset of public databases directly relevant to DTI prediction according to our survey (Table I). Brief description, data type, access link, and reference are also provided. A few databases will be referenced below for illustrative purposes.

### Curation Efforts

Valuable information on drugs, targets, and their associations usually scatter among literature and patent documents. Tremendous efforts have been devoted to literature curation, resulting in a great number of public databases, including DrugBank (18), KEGG (19), BRENDA (20), SuperTarget (21), STITCH (22), SIDER (24), ChEMBL (11), and BindingDB (25). As probably the most used resource, DrugBank contains comprehensive information about thousands of well-studied drugs and their targets. SIDER is a widely adopted database about marketed drugs and their recorded adverse drug reactions. In particular, Yamanishi et al. compiled a dataset (41) from DrugBank, KEGG, BRENDA, and SuperTarget on four major therapeutic target classes (i.e., enzyme, ion channel, G-protein coupled receptor, and nuclear receptor), which subsequently became a golden standard for DTI modeling. Many algorithms were built upon this dataset or its derivatives (see Supplementary Table S1). While most curated databases contain relatively small-scale and specialized data, ChEMBL has very large-scale bioactivity data, which were manually extracted from over 60,000 publications in medicinal chemistry literature. Additionally, the BindingDB project has recently started to curate chemical biology data in patent documents.

### Screening Programs and Open Repositories

Large-scale chemical biology data only became publicly available in the recent decade with the advent of several important screening programs and data repositories including NCI/DTP (26), Connectivity Map (CMap) (36), and PubChem BioAssay (8-10). The NCI/DTP is one of the first screening programs including the well-studied NCI human tumor cell line anticancer drug screen (NCI-60) dataset. CMap is a well-established resource on gene expression profiles induced by chemical perturbation in cell lines (36). The establishment of the PubChem project (https://pubchem.ncbi.nlm.nih.gov/) marks a milestone of open access to millions of biological test results of small molecules from HTS experiments. Unlike most curation projects, which usually contain only active data, PubChem serves as a public chemical biology data repository, which also archives inactive data from biological assays. This is crucial because accurate and robust predictive models depend on reliable negative samples as well. Moreover, the PubChem BioAssay database is growing rapidly with data deposited by worldwide researchers (Fig. 2) and becomes a hub for integrating chemical biological data resources including the abovementioned ChEMBL and NCI/DTP. As a data archiving and sharing system, a particular feature of PubChem BioAssay is that one can easily aggregate bioactivity data from multiple depositions for a specific target or drug, which could be beneficial for DTI prediction and evaluation purposes.

## APPROACHES

The expanding data accessibility has greatly facilitated innovative development for DTI prediction. In this review, we survey recent approaches and applications that were published in the last 5 years as well as a few earlier pioneering studies. Traditional methods like QSAR, pharmacophore modeling, and molecular docking were excluded. We obtained a list of over 80 studies aiming at DTI prediction, suggesting strong and growing research interest from the community. A concise summary of these studies is presented in Supplementary Table S1. The primary modeling method for each study has also been described briefly, although the discussion of technical details is beyond our scope.

Most studies for DTI prediction were based on the hypothesis that similar targets interact with same drug, and the same target interacts with similar drugs. The similarities among drugs reflect a chemical space while similarities among targets reflect a genomic space. These similarities play a key role and can be derived from various types of data. Our survey shows that the target similarities are mostly obtained on the basis of genomic sequence, e.g., sequence similarity by structural and physicochemical features or by sequence alignment score, though a few other target features were also used (42), including biological function (43), domain annotation (44), and proximity in the protein-protein interaction network (45). In comparison, data from the drug perspective is much more diverse as shown below. Due to page limitation, only a few applications will be described.

### Drug-Target Interaction

A set of known DTIs is required by any approach to build and/or evaluate models. Interestingly, using only the connections among drugs and targets, it is possible to make novel predictions via graph theory, network analysis, matrix factorization, etc. For instance, van Laarhoven et al. introduced the Gaussian interaction profile (GIP) kernel (46) and showed that it is capable of predicting true interaction pairs with high accuracy. Based on the complex network theory, Cheng et al. demonstrated that the network-based inference (NBI) performed best on the benchmark datasets (47). Cobanoglu et al. developed an active learning method with probabilistic matrix factorization (PMF), which is particularly useful for analyzing large interaction networks (48) because it is independent of chemical, structural, or other similarity metrics and its computation time scales are linear with the number of known interactions.

### Chemical Similarity

It is probably the most intuitive approach to predict novel DTIs for a query drug from a similar drug with known targets. Chemical similarity between two drugs can be defined by various means, e.g., based on sub-structural features or physicochemical descriptors, which can be calculated by popular software (Supplementary Table S2). A wide range of chemical descriptors were lately benchmarked in the context of DTI prediction (49). Recently, the SMILES-based compound similarity functions were proposed (50), which were found to be comparable to 13 other more computationally demanding similarity measures. It is noteworthy that one should use isomeric SMILES in order to handle stereochemistry correctly. The similarity ensemble approach (SEA) (51), developed by the Shoichet group, is a pioneering method for drug repositioning relying on two-dimensional (2D) similarity. A recent application of SEA from the same group (52) revealed that SEA can suggest structurally dissimilar compounds for a given target, although the similarity measure behind the scene is 2D. An extension to SEA was proposed recently by Zheng et al., termed weighted ensemble similarity (WES) (53). There are also other SEA-like approaches, e.g., SuperPred (54) and similarity ranking with data fusion (55).

While most predictive approaches for DTIs utilized 2D-based similarity given its lower computational cost, three-dimensional (3D) chemical similarities have demonstrated their strengths not seen in 2D similarity methods. For instance, AbdulHameed et al. presented a shape-based target fishing approach by using the ROCS program to generate 3D profiles for a set of drugs against a given target (56). Their method can successfully identify off-targets and also highlight the fact that the 3D-based method facilitates enrichment even for compounds which are not found to be similar in 2D. ChemMapper (57) is another approach for exploring target pharmacology using SHAFTS as the 3D similarity calculation method.

### Bioactivity Profile

The availability of chemical biology data across multiple assays for a common compound library enables the generation of bioactivity profiles, which can be informative for predicting DTIs. For example, Cheng et al. developed a bioactivity profile similarity search (BASS) method for associating targets to small molecules by using the known target annotations of related compounds (58). BASS was able to identify a significant fraction of structurally diverse compounds with similar bioactivities, indicating its capability of “scaffold hopping.” In another study, Vilar et al. (59) calculated the target interaction profile fingerprint (TIPF) based on the activity data from ChEMBL, as a binary vector of presence or absence of interaction with an array of targets. TIPF was further verified through molecular docking and experimental assays. Based on the HTS data in PubChem BioAssay, Helal et al. generated the comprehensive bioactivity profiles (PubChem HTSFPs) for more than 300,000 small molecules with bioactivity data from 243 different bioassays (60). By using PubChem HTSFPs as molecular descriptors, the authors achieved a 27-time improvement in hit expansion experiments. It was also found that PubChem HTSFPs retrieved hits that are structurally diverse and distinct from active compounds obtained by chemical similarity-based methods.

### Drug Side Effect

Side effects, or the adverse effects of drugs, contain important clinical phenotypic information that may be useful for predicting novel targets of a drug (61) and have been explored in relating drug-protein interaction network (62). Takarabe et al. developed a pharmacogenomic approach for predicting DTI by using the adverse event reporting system (AERS) from the US Food and Drug Administration (FDA). The authors demonstrated that the approach could predict unknown DTIs which cannot be predicted by drug chemical structure-based approaches (63). Most recently, side effect profiles have been applied to explore the similarities shared between antidepressants and immune-modulators, revealing potential novel targets for treating major depressive disorders (64). Drug side effects have also been incorporated as an important information source in various other studies for DTI prediction (65-69).

### Therapeutic Effect

The Anatomical Therapeutic Chemical (ATC) classification system categorizes drugs by their therapeutic and chemical characteristics. Cheng et al. (65) proposed the drug therapeutic similarity inference (DTSI) method by using the ATC code. The DTSI methods were found to be comparable to a drug structural similarity inference (DSSI) method and a drug side effect similarity inference (DSESI) method reported in the same work. Shi et al. (66) enhanced the drug similarity metric by including the non-structural ATC-based similarity, which performed better than previous measures. In combination with an eigenvalue transformation technique (70), the ATC taxonomy similarity between drugs was computed using a semantic similarity algorithm and used as one drug similarity metric. Likewise, disease terms related to drugs can be applied to evaluate the drug similarity via terminology metrics as shown in the semantics-based edge partitioning approach (semEP) for DTI prediction (71).

### Drug-Induced Gene Expression

Gene expression profiles arising from drug treatment can provide insights to DTI prediction. Our survey shows that CMap is the mostly used resource in this regard. To name a few examples, a transcriptomic approach (72) based on the drug-induced gene expression data in CMap with a machine learning classification technique was developed. It was observed that this approach can predict target proteins independent of data on compound chemical structures. Compound profile correlations from CMap can be utilized to create a drug network with densely connected nodes, which were then used by Jaeger et al. in a graph-based model to predict causal targets (73). In the work of Fakhraei et al., the Spearman rank correlation coefficient of gene expression responses to drugs retrieved from CMap was applied as one similarity measure between drugs (43).

### Drug Binding Site

Ligand-target interactions are mainly determined by the physicochemical properties of the binding sites, which also largely depend on the ligand substructures. A fragment interaction model (FIM) was proposed to describe the interactions between ligands and targets (74) by using the binding sites of the target-ligand complexes extracted from the sc-PDB database. The FIM method has the potential capability of molecular interpretation of ligand-target binding. In another study, Cao et al. (75) extracted 3D binding information from complex structure, either experimental result or theoretical model, according to well-established geometric criteria for a series of important interactions, such as H-bond, ionic interaction, π-π stacking, and non-polar contact. These 3D interactions were transformed into a one-dimensional (1D) binary string, named ligand-based interaction fingerprint (LIFt), which was able to recognize most of the native targets for the promiscuous kinase inhibitor staurosporine on the basis of experimentally determined complex structures. Meslamani et al. (76) found that an SVM classifier with a 3D-binding site kernel significantly outperformed a sequence-based target kernel in discriminating target-ligand PDB complexes from false pairs.

### Drug-Drug Interaction

Kim and co-workers proved that drug-drug interaction (DDI) is a promising feature for predicting DTIs (77). They collected two sources of DDI data, i.e., adverse DDI effect from drugs.com and pharmacological DDI from STITCH. The former is a modification of the effect of drugs when other drugs are co-administered, and the latter is a relation between compounds that is derived from similar activities. The accuracies of DPIs prediction using DDI were compared to those obtained using chemical structure and side effects data, indicating that DDI information contributed most to DTI prediction based on two machine learning methods.

### Ontology and Semantic Data

Drug similarity can be measured by ontological terms in a hierarchical classification. Based on the ChEBI ontology, Gao et al. developed a model for identifying the target group for given drugs (78). Though this method does not predict DTIs explicitly, it is helpful for deducing the potential drug target within those target groups. The ChEBI ontology was also used by Chen et al. as one data source in their semantic annotated network (79). The authors developed the semantic link association prediction (SLAP) algorithm for predicting “missing links” in the network. Using an ontology-based data representation of the relationships among drugs, diseases, genes, pathways, and SNPs, Tao et al. successfully identified potential targets for colorectal cancer drugs through semantic reasoning (80).

### Literature and Text Mining

Hidden DTIs in literature can be discovered via text mining based on co-occurrence of drug and target entities. One pioneer study is from Zhu et al., in which the authors developed a probabilistic model, called the mixture aspect model (MAM), for mining implicit “chemical compound-gene” relations from the MEDLINE records (81). Recently, a new text mining technique was proposed by Geethanjali et al. (82) that can estimate the point-wise mutual information (PMI) among protein names obtained from UniProtKB and the Medical Subject Headings (MeSH) that contain drug terms extracted from MEDLINE. Based on PMI scores, gene/protein profiles and drug were produced and candidate drug-gene/protein associations were constructed when evaluating the relevance of their profiles.

### Quantitative Bioactivity Data

Most DTI prediction approaches have not taken full advantage of the quantitative bioactivity data provided in many chemical biology datasets; instead, only the true/false association of a DTI was used based on an activity threshold, usually 10 μM. In a recent work, Sugaya transformed the activity data from ChEMBL into binding efficiency index (BEI) (83). The SVM classifiers from the BEI-based training data demonstrated slightly higher performance in the cross-validation tests. Using a modified version of the influence-relevance voter (PS-IRV), Lusci et al. (84) showed that target prediction can be improved by making use of bioactivity data, where a compound was assigned different weights according to its potency range. In the work of Wang et al. (85), multiple types of DTIs (e.g., activation, inhibition, and binding) were differentiated. Their approach, called restricted Boltzmann machine, was able to predict drug mode of action in addition to DTIs.

## ONLINE TOOLS

Stimulated by the growing interest in DTI study and the availability of open data resources, many online tools have now been provided with open-access for DTI prediction including SEA (51), SuperPred (54), and ChemMapper (57). These tools may be readily used for DTI prediction without the need of a comprehensive understanding for the mathematical and computational complexity, hence greatly lower the barrier of collaborations among researchers across multiple disciplines. Undoubtedly, having easy access to tools for large-scale data analysis plays an important role in the era of big data for supporting data science. More online tools can be found in Supplementary Table S3 together with brief descriptions about algorithms, data types and additional information.

## DISCUSSION

### Data Integration and Data Fusion

The studies described above were purposely categorized according to individual types of data. Nevertheless, it is extremely important to integrate data from multiple sources and categories, and indeed, various sources of data were often combined in practice. For example, Wang et al. (69) calculated drug similarities on the basis of molecular structure, pharmacological information from the JAPIC database, therapeutic information from ATC code, side effects from the SIDER database, and activity data with target proteins from multiple sources, respectively. With these drug-related omics data, the authors concluded that data integration did help to improve DTI prediction. Moreover, data integration is not limited to drug centric information. Protein sequence information were also incorporated in their study. Actually, most applications in our survey make use of both drug and target data to a certain extent in chemogenomic methods. In a semantic network, Chen et al. (79) included different annotations relating to drugs, chemical compounds, protein targets, diseases, side effects, and pathways from 15 public databases, demonstrating the great potential of semantic network for integrating complex and heterogeneous data. In addition, data fusion is commonly observed in many approaches. In the chemical similarity ensemble approach (86), Wang et al. combined several SEA models, each employing a different fingerprint/descriptor (i.e., Morgan, atom pair, topological torsions, MACCS keys, 2D pharmacophore fingerprint, and SHED), which can be calculated from chemical structures. The ensemble version was found to outperform individual SEA models. There are also applications of data fusion using target information (42). Therefore, data integration using distinct and complementary source and data fusion through ensemble learning will continue to be promising approaches in the future.

### Data Imbalance and Negative Samples

One key challenge in DTI prediction is that the number of experimentally verified DTIs is relatively small. The fact that negative DTIs dominate over positive ones creates a known issue named “data imbalance”. This is especially critical to supervised learning, where models built with imbalanced data are prone to be biased toward major classes (i.e., negative DTIs), leading to more false negatives and thus may miss important DTIs. Common strategies to address this issue include random sampling, down sampling, over sampling, and balanced sampling (87). However, the resulting dataset may become unreliable due to data redundancy and/or information loss. Moreover, negative DTIs may not be reliable in the first place. Known DTIs were primarily curated from literature, which hardly report negative DTIs. As a consequence, researchers have little choice other than treating all unverified DTIs as negative samples despite that some of them may be true DTIs. Several recent applications were proposed to tackle this problem by treating non-interaction pairs as unlabeled (88,89), building up highly credible negative samples (44), or class imbalance-aware ensemble learning (90). Nevertheless, these techniques may be overly simplified according to a recent review (17). Therefore, much room is left along this direction. One possible strategy is to take advantage of the negative samples reported in large repositories, for example, the HTS data in PubChem. Figure 2 highlights that over 150 million negative CPIs are publicly available in PubChem BioAssay, which were experimentally verified and could be beneficial for building more accurate predictive models. One recent application from Fu et al. made use of the PubChem BioAssay data to negatively label about 800,000 out of a total of 5.6 billion links in their semantic network (91).

### Data Availability and Cross-Linking

DTI prediction in a “big data” era creates both opportunities and challenges. The increasing availability of data has dramatically stimulated the development of novel DTI prediction methods. On the other hand, there are many data types that are still not adequately available; also, the lower data quality may lead to inaccurate prediction. In fact, our knowledge regarding the entire chemogenomic space is far from comprehensive. As an example, only half a million compounds out of the total 90 million unique chemicals registered in PubChem are associated with CPI data (Fig. 2). Furthermore, the widely applied pharmacological and therapeutic data (e.g., side effect and ATC code) are very sparse and difficult to obtain. In addition, bioactivity profile and gene expression are costly to produce. 3D protein structures and thus the binding sites, which are essential for the profound understanding of DTIs, are largely unavailable for some important therapeutic targets including membrane proteins. Besides data availability, another major issue is the varying data quality among different data types. Data generated from HTS experiments are known to be noisy and possibly contain artifacts. Inconsistency may occur when incorporating bioactivity data from multiple experiments for the same chemicals regardless of experimental conditions, warning that data quality control should be taken into account when applying data for DTI prediction.

It has been demonstrated that the utility of integrated data sources helps to improve DTI prediction. Computer-readable cross-links among different sources of biological data thus play a key role for data integration and information discovery. However, hurdles still exist due to the lack of cross-references among data sources which are highly relevant but generated by different research communities. For instance, gene expression data provides valuable information for deducting gene targets when small-molecule drugs were used for perturbation of the cell system. However, drug molecule information in a gene expression data repository may be limited to a chemical name and may be stored simply in a textual context; hence, the lack of accurate linking to chemical structure data in a public chemical database makes it difficult to combine widely available gene expression data on a large scale for DTI prediction.

Despite the lack of communications and linking for data resources across research communities, there are encouraging progresses. Recently, the Findability, Accessibility, Interoperability, and Reusability (FAIR) principle has been proposed to provide guidance for managing public data, maintaining data flow, and sharing analysis tools and pipelines (92). This effort is to bring clarity and encourage public data stakeholders to work toward the simple guidance together with funding agency, researcher, and publisher to harmonize research data. Identifiers of genomic data, such as accession of a nucleotide sequence, have been required for submission of PubMed. Open-access journals, such as those from Elsevier, require the provision of chemical identifiers in PubChem (e.g., unique chemical structure accession, CID) for chemicals reported in the publication. Government funding agencies and journal publishers are required to take further steps toward open access and data sharing. Synergized efforts from researchers of multiple disciplines in support of open data and data science are needed and would greatly help to develop novel system biology methodologies and accelerate discoveries.

## SUMMARY

We have reviewed public databases, online tools, and recent applications relevant to DTI prediction from the data perspective. It is found that various types of data were employed for in silico studies, such as chemical structure, bioactivity profile, side effect, therapeutic effect, drug-induced gene expression, drug binding site, drug-drug interaction, and ontology and semantic data. More often, the heterogeneous data were integrated to offer boosted performance. Given such an important field in drug discovery, we anticipate more advances to come along with the growing availability of chemogenomic data and innovations in computation power. However, many challenges remain with respect to data accessibility, process, and analysis. Better strategies for dealing with data imbalance and incorporating negative samples are desired. Full utility of quantitative bioactivity data remain to be explored. Multiple dimensional and high-quality data as well as open-access online tools supporting data analysis are in great need. Additionally, it also remains a great challenge for public data repositories, database stakeholders, and journal publishers to work harmoniously for producing and maintaining cross-links among data generated from different scientific disciplines.

## Supplementary Material

Supplementary Table S1, S2 and S3

## Figures and Tables

**Table I.** Public Databases Relevant for Predicting Drug-Target Interactions

Resource	Description	Data content	Website	Ref.
Curated drug-target interactions				
DrugBank	A comprehensive resource that combines detailed drug (i.e., chemical, pharmacological and pharmaceutical) data with comprehensive drug target (i.e., sequence, structure, and pathway) information	8206 drug entries including 1991 FDA-approved small-molecule drugs, 207 FDA-approved biotech drugs, 93 nutraceuticals and over 6000 experimental drugs;	http://www.drugbank.ca/	(18)
ChEMBL	A manually curated chemical database of bioactive molecules with drug-like properties	11,019 targets; 1,592,191 compounds; 13,967,816 activities	https://www.ebi.ac.uk/chembldb/	(11)
KEGG	A comprehensive resource, including drugs, genes, reactions, pathways, and diseases, for understanding high-level functions and utilities of the biological system	17,840 compounds; 10,431 drugs; 20,620,439 genes	http://www.kegg.jp/kegg/	(19)
Comparative Toxicogenomics Database (CTD)	A public website and research tool that curates scientific data describing relationships between chemicals/drugs, genes/proteins, diseases, taxa, phenotypes, GO annotations, pathways, and interaction modules	1,379,105 chemical-gene interactions; 19,753,624 gene-disease associations; 2,060,371; 14,672 chemicals; 6401 diseases; 42,761 genes	http://ctdbase.org/	(27)
Guide to PHARMACOLOGY	An open-access website, acting as a portal to information on the biological targets of licensed drugs and other small molecules	2789 targets; 8611 ligands; 14,577 curated binding constants; 31,207 binding constants from large-scale screening	http://www.guidetopharmacology.org/	(28)
Therapeutic Target Database (TTD)	A database to provide information about the known and explored therapeutic protein and nucleic acid targets, the targeted disease, pathway information, and the corresponding drugs directed at each of these targets	2025 target; 17,816 drugs	http://bidd.nus.edu.sg/group/cjttd/	(23)
STITCH	A resource to explore known and predicted interactions of chemicals and proteins	300,000 small molecules; 2.6 million proteins; 1133 organisms	http://stitch.embl.de/	(22)
SuperTarget	An extensive web resource for analyzing drug-target interactions	6219 targets; 195,770 compounds; 332,828 drug-target interactions; 282 drug-target related pathways; 6532 drug-target related ontologies; 63 cytochromes	http://insilico.charite.de/supertarget/	(21)
DrugKiNET	An open-access, online resource to foster the identification and characterization of inhibitors of protein kinases for academic and industrial research	400 human kinases; 800 inhibitors; 105,000 kinase-compound pairs	http://www.drugkinet.ca/	
PROMISCUOUS	An exhaustive resource of protein-protein and drug-protein interactions with the aim of providing a uniform dataset for drug repositioning and further analysis	5258 drugs with targets; 6548 targets with drugs; 23,702 drug-target interactions	http://bioinformatics.charite.de/promiscuous/	(29)
NCGC Pharmaceutical Collection	A comprehensive resource of clinically approved drugs enabling repurposing and chemical genomics	14,814 compounds; 1270 targets	https://tripod.nih.gov/npc/	(30)
ChemProt	A resource of annotated and predicted chemical-protein interactions	1.7 million chemicals; 20,000 proteins	http://potentia.cbs.dtu.dk/ChemProt/	(37)
BRENDA	The main enzyme and enzyme-ligand information system	83,000 enzymes; 206,000 enzyme ligands	http://www.brenda-enzymes.org/	(20)
MATADOR	A manually annotated targets and drug online resource	1500 drugs; 2500 target proteins; 7300 drug-protein elations	http://matador.embl.de/	(39)
3D structures and/or binding affinities				
Protein Data Bank (PDB)	A crystallographic database for the three-dimensional structural data of large biological molecules, such as proteins and nucleic acids	120,642 biological macromolecular structures	http://www.rcsb.org/pdb/home/home.do	(31)
MMDB	A collection of publicly accessible experimentally determined macromolecular structures with added information on the biological function and the evolutionary history of macromolecules	119,566 macromolecular structures, with 86,960 contains chemicals	https://www.ncbi.nlm.nih.gov/structure	(32)
PDBbind	A comprehensive collection of the experimentally measured binding affinity data for all types of biomolecular complexes deposited in the Protein Data Bank (PDB)	14,620 biomolecular complexes, including protein-ligand (11,987), nucleic acid-ligand (109), protein-nucleic acid (717), and protein-protein complexes (1807)	http://www.pdbbind-cn.org/	(33)
BindingDB	A public, web-accessible database of measured binding affinities, focusing chiefly on the interactions of proteins considered to be candidate drug targets with ligands that are small, drug-like molecules	1,242,569 binding data; 6395 protein targets, 547,013 small molecules	http://bindingdb.org/	(25)
Binding MOAD	A collection of well-resolved protein crystal structures with clearly identified biologically relevant ligands annotated with experimentally determined binding data extracted from literature	25,771 protein-ligand structures; 9142 binding data; 12,440 different ligands; 7599 different families	http://bindingmoad.org/	(34)
PDSP Ki database	A unique resource in the public domain which provides information on the abilities of drugs to interact with an expanding number of molecular targets	59,705 Ki values	http://kidbdev.med.unc.edu/databases/pdsp.php	(40)
Side effects				
SIDER	An information portal on marketed medicines and their recorded adverse drug reactions	1430 drugs; 5868 side effects; 139,756 drug-side effect pairs	http://sideeffects.embl.de/	(24)
FAERS	FDA’s adverse event reporting system		http://www.fda.gov/Drugs/GuidanceComplianceRegulatoryInformation/Surveillance/AdverseDrugEffects/default.htm	
MetaADEDB	A comprehensive computer-available adverse drug events database	3060 chemicals; 13,256 side effects; 527,216 drug-ADEs associations	http://lmmd.ecust.edu.cn/online_services/metaadedb/	(38)
JAPIC	Side effects		http://www.japic.or.jp/	
Large screening programs and data repositories				
PubChem BioAssay	A public repository for bioactivity data of small molecules and RNAi reagents against thousands of molecular targets	1.2 million bioassays; 3 million tested substances (2 million compounds); 250 million bioactivities; 10,000 protein targets	https://ncbi.nlm.nih.gov/pcassay/	(8) (9) (10)
NCI/DTP	The Developmental Therapeutics Program of the National Cancer Institute		https://dtp.cancer.gov/	(26)
ChemBank	A public, web-based informatics environment for data derived from small molecules and small-molecule screens		http://chembank.broadinstitute.org/	(35)
Connectivity Map (CMap)	A catalog of gene expression data collected from human cells treated with chemical compounds and genetic reagents	6100 gene expression profiles; 13,469 human genes; 1309 bioactive small compounds	https://www.broadinstitute.org/connectivity-map-cmap	(36)

## References

[1] Ashburn TT, Thor KB. Drug repositioning: identifying and developing new uses for existing drugs. Nat Rev Drug Discov. 2004;3(8):673–83. 15286734 10.1038/nrd1468

[2] Day D, Siu LL. Approaches to modernize the combination drug development paradigm. Genome Med. 2016;8(1):115. 27793177 10.1186/s13073-016-0369-x PMC5084460

[3] Medina-Franco JL, Giulianotti MA, Welmaker GS, Houghten RA. Shifting from the single to the multitarget paradigm in drug discovery. Drug Discov Today. 2013;18(9–10):495–501. 23340113 10.1016/j.drudis.2013.01.008 PMC3642214

[4] Keiser MJ, Setola V, Irwin JJ, Laggner C, Abbas AI, Hufeisen SJ, Predicting new molecular targets for known drugs. Nature. 2009;462(7270):175–81. 19881490 10.1038/nature08506 PMC2784146

[5] Wang T, Wu MB, Lin JP, Yang LR. Quantitative structure-activity relationship: promising advances in drug discovery platforms. Expert Opin Drug Discov. 2015;10(12):1283–300. 26358617 10.1517/17460441.2015.1083006

[6] Yang SY. Pharmacophore modeling and applications in drug discovery: challenges and recent advances. Drug Discov Today. 2010;15(11–12):444–50. 20362693 10.1016/j.drudis.2010.03.013

[7] Cheng T, Li Q, Zhou Z, Wang Y, Bryant SH. Structure-based virtual screening for drug discovery: a problem-centric review. AAPS J. 2012;14(1):133–41. 22281989 10.1208/s12248-012-9322-0 PMC3282008

[8] Wang Y, Bryant SH, Cheng T, Wang J, Gindulyte A, Shoemaker BA, PubChem BioAssay: 2017 update. Nucleic Acids Res. 2017;45(D1):D955–D63. 27899599 10.1093/nar/gkw1118 PMC5210581

[9] Wang Y, Suzek T, Zhang J, Wang J, He S, Cheng T, PubChem BioAssay: 2014 update. Nucleic Acids Res. 2014;42(Database issue):D1075–82. 24198245 10.1093/nar/gkt978 PMC3965008

[10] Wang YL, Xiao JW, Suzek TO, Zhang J, Wang JY, Zhou ZG, PubChem’s bioassay database. Nucleic Acids Res. 2012;40(D1):D400–D12. 22140110 10.1093/nar/gkr1132 PMC3245056

[11] Bento AP, Gaulton A, Hersey A, Bellis LJ, Chambers J, Davies M, The ChEMBL bioactivity database: an update. Nucleic Acids Res. 2014;42(D1):D1083–D90. 24214965 10.1093/nar/gkt1031 PMC3965067

[12] Chen X, Yan CC, Zhang X, Zhang X, Dai F, Yin J, Drug-target interaction prediction: databases, web servers and computational models. Brief Bioinform. 2016;17(4):696–712. 26283676 10.1093/bib/bbv066

[13] Ding H, Takigawa I, Mamitsuka H, Zhu S. Similarity-based machine learning methods for predicting drug-target interactions: a brief review. Brief Bioinform. 2014;15(5):734–47. 23933754 10.1093/bib/bbt056

[14] Li J, Zheng S, Chen B, Butte AJ, Swamidass SJ, Lu Z. A survey of current trends in computational drug repositioning. Brief Bioinform. 2016;17(1):2–12. 25832646 10.1093/bib/bbv020 PMC4719067

[15] Dai YF, Zhao XM. A survey on the computational approaches to identify drug targets in the postgenomic era. Biomed Res Int. 2015;2015:239654. 26060814 10.1155/2015/239654 PMC4427773

[16] Mousavian Z, Masoudi-Nejad A. Drug-target interaction prediction via chemogenomic space: learning-based methods. Expert Opin Drug Metab Toxicol. 2014;10(9):1273–87. 25112457 10.1517/17425255.2014.950222

[17] Pahikkala T, Airola A, Pietila S, Shakyawar S, Szwajda A, Tang J, Toward more realistic drug-target interaction predictions. Brief Bioinform. 2015;16(2):325–37. 24723570 10.1093/bib/bbu010 PMC4364066

[18] Law V, Knox C, Djoumbou Y, Jewison T, Guo AC, Liu Y, DrugBank 4.0: shedding new light on drug metabolism. Nucleic Acids Res. 2014;42(D1):D1091–D7. 24203711 10.1093/nar/gkt1068 PMC3965102

[19] Kanehisa M, Furumichi M, Tanabe M, Sato Y, Morishima K. KEGG: new perspectives on genomes, pathways, diseases and drugs. Nucleic Acids Res. 2017;45(D1):D353–D61. 27899662 10.1093/nar/gkw1092 PMC5210567

[20] Placzek S, Schomburg I, Chang A, Jeske L, Ulbrich M, Tillack J, BRENDA in 2017: new perspectives and new tools in BRENDA. Nucleic Acids Res. 2017;45(D1):D380–D8. 27924025 10.1093/nar/gkw952 PMC5210646

[21] Hecker N, Ahmed J, von Eichborn J, Dunkel M, Macha K, Eckert A, SuperTarget goes quantitative: update on drug-target interactions. Nucleic Acids Res. 2012;40(Database issue):D1113–7. 22067455 10.1093/nar/gkr912 PMC3245174

[22] Kuhn M, Szklarczyk D, Pletscher-Frankild S, Blicher TH, von Mering C, Jensen LJ, STITCH 4: integration of protein-chemical interactions with user data. Nucleic Acids Res. 2014;42(Database issue):D401–7. 24293645 10.1093/nar/gkt1207 PMC3964996

[23] Yang H, Qin C, Li YH, Tao L, Zhou J, Yu CY, Therapeutic target database update 2016: enriched resource for bench to clinical drug target and targeted pathway information. Nucleic Acids Res. 2016;44(D1):D1069–D74. 26578601 10.1093/nar/gkv1230 PMC4702870

[24] Kuhn M, Letunic I. The SIDER database of drugs and side effects. 2016;44(D1):D1075–9. 10.1093/nar/gkv1075 PMC4702794 26481350

[25] Gilson MK, Liu T, Baitaluk M, Nicola G, Hwang L, Chong J. BindingDB in 2015: a public database for medicinal chemistry, computational chemistry and systems pharmacology. Nucleic Acids Res. 2016;44(D1):D1045–D53. 26481362 10.1093/nar/gkv1072 PMC4702793

[26] Shoemaker RH. The NCI60 human tumour cell line anticancer drug screen. Nat Rev Cancer. 2006;6(10):813–23. 16990858 10.1038/nrc1951

[27] Grondin CJ, Davis AP, Wiegers TC, King BL, Wiegers JA, Reif DM, Advancing exposure science through chemical data curation and integration in the comparative Toxicogenomics database. Environ Health Perspect. 2016;124(10):1592–9. 27170236 10.1289/EHP174 PMC5047769

[28] Southan C, Sharman JL, Benson HE, Faccenda E, Pawson AJ, Alexander SP, The IUPHAR/BPS guide to PHARMACOLOGY in 2016: towards curated quantitative interactions between 1300 protein targets and 6000 ligands. Nucleic Acids Res. 2016;44:D1054–D68. 26464438 10.1093/nar/gkv1037 PMC4702778

[29] von Eichborn J, Murgueitio MS, Dunkel M, Koerner S, Bourne PE, Preissner R. PROMISCUOUS: a database for network-based drug-repositioning. Nucleic Acids Res. 2011;39(Database issue):D1060–6. 21071407 10.1093/nar/gkq1037 PMC3013657

[30] Huang R, Southall N, Wang Y, Yasgar A, Shinn P, Jadhav A, The NCGC pharmaceutical collection: a comprehensive resource of clinically approved drugs enabling repurposing and chemical genomics. Sci Transl Med. 2011;3(80):80ps16. 10.1126/scitranslmed.3001862 PMC3098042 21525397

[31] HM Berman, Westbrook J, Feng Z, Gilliland G, Bhat TN, Weissig H, The protein data bank. Nucleic Acids Res. 2000;28(1):235–42. 10592235 10.1093/nar/28.1.235 PMC102472

[32] Madej T, Lanczycki CJ, Zhang D, Thiessen PA, Geer RC, Marchler-Bauer A, MMDB and VAST+: tracking structural similarities between macromolecular complexes. Nucleic Acids Res. 2014;42:D297–303. 24319143 10.1093/nar/gkt1208 PMC3965051

[33] Liu Z, Li Y, Han L, Li J, Liu J, Zhao Z, PDB-wide collection of binding data: current status of the PDBbind database. Bioinformatics. 2015;31(3):405–12. 25301850 10.1093/bioinformatics/btu626

[34] Ahmed A, Smith RD, Clark JJ, Dunbar JB Jr, Carlson HA. Recent improvements to binding MOAD: a resource for protein-ligand binding affinities and structures. Nucleic Acids Res. 2015;43(Database issue):D465–9. 25378330 10.1093/nar/gku1088 PMC4383918

[35] Seiler KP, George GA, Happ MP, Bodycombe NE, Carrinski HA, Norton S, ChemBank: a small-molecule screening and cheminformatics resource database. Nucleic Acids Res. 2008;36(Database issue):D351–9. 17947324 10.1093/nar/gkm843 PMC2238881

[36] Lamb J, Crawford ED, Peck D, Modell JW, Blat IC, Wrobel MJ, The connectivity map: using gene-expression signatures to connect small molecules, genes, and disease. Science. 2006;313(5795):1929–35. 17008526 10.1126/science.1132939

[37] Kringelum J, Kjaerulff SK, Brunak S, Lund O, Oprea TI, Taboureau O. ChemProt-3.0: a global chemical biology diseases mapping. Database. 2016;bav123. 26876982 10.1093/database/bav123 PMC4752971

[38] Cheng F, Li W, Wang X, Zhou Y, Wu Z, Shen J, Adverse drug events: database construction and in silico prediction. J Chem Inf Model. 2013;53(4):744–52. 23521697 10.1021/ci4000079

[39] Gunther S, Kuhn M, Dunkel M, Campillos M, Senger C, Petsalaki E, SuperTarget and Matador: resources for exploring drug-target relationships. Nucleic Acids Res. 2008;36(Database issue):D919–22. 17942422 10.1093/nar/gkm862 PMC2238858

[40] Roth BL, Lopez E, Patel S, Kroeze WK. The multiplicity of serotonin receptors: uselessly diverse molecules or an embarrassment of riches? Neuroscientist. 2000;6(4):252–62.

[41] Yamanishi Y, Araki M, Gutteridge A, Honda W, Kanehisa M. Prediction of drug-target interaction networks from the integration of chemical and genomic spaces. Bioinformatics. 2008;24(13):i232–i40. 18586719 10.1093/bioinformatics/btn162 PMC2718640

[42] Nanni L, Lumini A, Brahnam S. A set of descriptors for identifying the protein-drug interaction in cellular networking. J Theor Biol. 2014;359:120–8. 24949993 10.1016/j.jtbi.2014.06.008

[43] Fakhraei S, Raschid L, Getoor L, editors. Drug-target interaction prediction for drug repurposing with probabilistic similarity logic. Proceedings of the 12th international workshop on data mining in bioinformatics. ACM; 2013.

[44] Liu H, Sun J, Guan J, Zheng J, Zhou S. Improving compound-protein interaction prediction by building up highly credible negative samples. Bioinformatics. 2015;31(12):i221–9. 26072486 10.1093/bioinformatics/btv256 PMC4765858

[45] Nascimento AC, Prudencio RB, Costa IG. A multiple kernel learning algorithm for drug-target interaction prediction. BMC Bioinformatics. 2016;17:46. 26801218 10.1186/s12859-016-0890-3 PMC4722636

[46] van Laarhoven T, Nabuurs SB, Marchiori E. Gaussian interaction profile kernels for predicting drug-target interaction. Bioinformatics. 2011;27(21):3036–43. 21893517 10.1093/bioinformatics/btr500

[47] Cheng F, Liu C, Jiang J, Lu W, Li W, Liu G, Prediction of drug-target interactions and drug repositioning via network-based inference. PLoS Comput Biol. 2012;8(5):e1002503. 22589709 10.1371/journal.pcbi.1002503 PMC3349722

[48] Cobanoglu MC, Liu C, Hu F, Oltvai ZN, Bahar I. Predicting drug-target interactions using probabilistic matrix factorization. J Chem Inf Model. 2013;53(12):3399–409. 24289468 10.1021/ci400219z PMC3871285

[49] Sawada R, Kotera M, Yamanishi Y. Benchmarking a wide range of chemical descriptors for drug-target interaction prediction using a chemogenomic approach. Mol Inform. 2014;33(11–12):719–31. 27485418 10.1002/minf.201400066

[50] Ozturk H, Ozkirimli E, Ozgur A. A comparative study of SMILES-based compound similarity functions for drug-target interaction prediction. BMC Bioinformatics. 2016;17:128. 26987649 10.1186/s12859-016-0977-x PMC4797122

[51] Keiser MJ, Roth BL, Armbruster BN, Ernsberger P, Irwin JJ, Shoichet BK. Relating protein pharmacology by ligand chemistry. Nat Biotechnol. 2007;25(2):197–206. 17287757 10.1038/nbt1284

[52] Lounkine E, Keiser MJ, Whitebread S, Mikhailov D, Hamon J, Jenkins JL, Large-scale prediction and testing of drug activity on side-effect targets. Nature. 2012;486(7403):361–7. 22722194 10.1038/nature11159 PMC3383642

[53] Zheng C, Guo Z, Huang C, Wu Z, Li Y, Chen X, Large-scale direct targeting for drug repositioning and discovery. Sci Rep. 2015;5:11970. 26155766 10.1038/srep11970 PMC4496667

[54] Nickel J, Gohlke BO, Erehman J, Banerjee P, Rong WW, Goede A, SuperPred: update on drug classification and target prediction. Nucleic Acids Res. 2014;42(Web Server issue):W26–31. 24878925 10.1093/nar/gku477 PMC4086135

[55] Liu X, Xu Y, Li S, Wang Y, Peng J, Luo C, In silico target fishing: addressing a “big data” problem by ligand-based similarity rankings with data fusion. J Cheminform. 2014;6:33. 24976868 10.1186/1758-2946-6-33 PMC4068908

[56] AbdulHameed MD, Chaudhury S, Singh N, Sun H, Wallqvist A, Tawa GJ. Exploring polypharmacology using a ROCS-based target fishing approach. J Chem Inf Model. 2012;52(2):492–505. 22196353 10.1021/ci2003544

[57] Gong J, Cai C, Liu X, Ku X, Jiang H, Gao D, ChemMapper: a versatile web server for exploring pharmacology and chemical structure association based on molecular 3D similarity method. Bioinformatics. 2013;29(14):1827–9. 23712658 10.1093/bioinformatics/btt270

[58] Cheng T, Li Q, Wang Y, Bryant SH. Identifying compound-target associations by combining bioactivity profile similarity search and public databases mining. J Chem Inf Model. 2011;51(9):2440–8. 21834535 10.1021/ci200192v PMC3180241

[59] Vilar S, Quezada E, Uriarte E, Costanzi S, Borges F, Vina D, Computational drug target screening through protein interaction profiles. Sci Rep. 2016;6:36969. 27845365 10.1038/srep36969 PMC5109486

[60] Helal KY, Maciejewski M, Gregori-Puigjane E, Glick M, Wassermann AM. Public domain HTS fingerprints: design and evaluation of compound bioactivity profiles from PubChem’s BioAssay repository. J Chem Inf Model. 2016;56(2):390–8. 26898267 10.1021/acs.jcim.5b00498

[61] Campillos M, Kuhn M, Gavin AC, Jensen LJ, Bork P. Drug target identification using side-effect similarity. Science. 2008;321(5886):263–6. 18621671 10.1126/science.1158140

[62] Mizutani S, Pauwels E, Stoven V, Goto S, Yamanishi Y. Relating drug-protein interaction network with drug side effects. Bioinformatics. 2012;28(18):i522–i8. 22962476 10.1093/bioinformatics/bts383 PMC3436810

[63] Takarabe M, Kotera M, Nishimura Y, Goto S, Yamanishi Y. Drug target prediction using adverse event report systems: a pharmacogenomic approach. Bioinformatics. 2012;28(18):i611–i8. 22962489 10.1093/bioinformatics/bts413 PMC3436840

[64] Sun Y, Narayan VA, Wittenberg GM. Side effect profile similarities shared between antidepressants and immune-modulators reveal potential novel targets for treating major depressive disorders. BMC Pharmacol Toxicol. 2016;17(1):47. 27765060 10.1186/s40360-016-0090-9 PMC5073882

[65] Cheng F, Li W, Wu Z, Wang X, Zhang C, Li J, Prediction of polypharmacological profiles of drugs by the integration of chemical, side effect, and therapeutic space. J Chem Inf Model. 2013;53(4):753–62. 23527559 10.1021/ci400010x

[66] Shi JY, Yiu SM, Li Y, Leung HC, Chin FY. Predicting drug-target interaction for new drugs using enhanced similarity measures and super-target clustering. Methods. 2015;83:98–104. 25957673 10.1016/j.ymeth.2015.04.036

[67] Vilar S, Hripcsak G. Leveraging 3D chemical similarity, target and phenotypic data in the identification of drug-protein and drug-adverse effect associations. J Cheminform. 2016;8:35. 27375776 10.1186/s13321-016-0147-1 PMC4930585

[68] Yamanishi Y, Kotera M, Moriya Y, Sawada R, Kanehisa M, Goto S. DINIES: drug-target interaction network inference engine based on supervised analysis. Nucleic Acids Res. 2014;42(Web Server issue):W39–45. 24838565 10.1093/nar/gku337 PMC4086078

[69] Wang YC, Deng N, Chen S, Wang Y. Computational study of drugs by integrating omics data with kernel methods. Mol Inform. 2013;32(11–12):930–41. 27481139 10.1002/minf.201300090

[70] Kuang Q, Xu X, Li R, Dong Y, Li Y, Huang Z, An eigenvalue transformation technique for predicting drug-target interaction. Sci Rep. 2015;5:13867. 26350590 10.1038/srep13867 PMC4563363

[71] Palma G, Vidal M-E, Raschid L, editors. Drug-target interaction prediction using semantic similarity and edge partitioning. International semantic web conference. Springer; 2014.

[72] Hizukuri Y, Sawada R, Yamanishi Y. Predicting target proteins for drug candidate compounds based on drug-induced gene expression data in a chemical structure-independent manner. BMC Med Genet. 2015;8:82. 10.1186/s12920-015-0158-1 PMC4683716 26684652

[73] Jaeger S, Min J, Nigsch F, Camargo M, Hutz J, Cornett A, Causal network models for predicting compound targets and driving pathways in cancer. J Biomol Screen. 2014;19(5):791–802. 24518063 10.1177/1087057114522690

[74] Wang C, Liu J, Luo F, Deng Z, Hu QN. Predicting target-ligand interactions using protein ligand-binding site and ligand substructures. BMC Syst Biol. 2015;9(Suppl 1):S2. 10.1186/1752-0509-9-S1-S2 PMC4331677 25707321

[75] Cao R, Wang Y. In silico study of polypharmacology with ligand-based interaction fingerprint. Receptors Clin Investig. 2015;2(4):e976.

[76] Meslamani J, Rognan D. Enhancing the accuracy of chemogenomic models with a three-dimensional binding site kernel. J Chem Inf Model. 2011;51(7):1593–603. 21644501 10.1021/ci200166t

[77] Kim S, Jin D, Lee H. Predicting drug-target interactions using drug-drug interactions. PLoS One. 2013;8(11):e80129. 24278248 10.1371/journal.pone.0080129 PMC3836969

[78] Gao YF, Chen L, Huang GH, Zhang T, Feng KY, Li HP, Prediction of drugs target groups based on ChEBI ontology. Biomed Res Int. 2013;2013:132724. 24350241 10.1155/2013/132724 PMC3853244

[79] Chen B, Ding Y, Wild DJ. Assessing drug target association using semantic linked data. PLoS Comput Biol. 2012;8(7):e1002574. 22859915 10.1371/journal.pcbi.1002574 PMC3390390

[80] Tao C, Sun J, Zheng WJ, Chen J, Xu H. Colorectal cancer drug target prediction using ontology-based inference and network analysis. Database. 2015;2015:bav015. 25818893 10.1093/database/bav015 PMC4375358

[81] Zhu S, Okuno Y, Tsujimoto G, Mamitsuka H. A probabilistic model for mining implicit ‘chemical compound-gene’ relations from literature. Bioinformatics. 2005;21 Suppl 2:ii245–51. 16204113 10.1093/bioinformatics/bti1141

[82] Geethanjali C, Bhanumathi S. Generating drug-gene association for Vibrio cholerae using ontological profile similarity. Indian J Sci Technol. 2016;9(33):99620.

[83] Sugaya N. Training based on ligand efficiency improves prediction of bioactivities of ligands and drug target proteins in a machine learning approach. J Chem Inf Model. 2013;53(10):2525–37. 24020509 10.1021/ci400240u

[84] Lusci A, Browning M, Fooshee D, Swamidass J, Baldi P. Accurate and efficient target prediction using a potency-sensitive influence-relevance voter. J Cheminform. 2015;7:63. 26719774 10.1186/s13321-015-0110-6 PMC4696267

[85] Wang Y, Zeng J. Predicting drug-target interactions using restricted Boltzmann machines. Bioinformatics. 2013;29(13):i126–34. 23812976 10.1093/bioinformatics/btt234 PMC3694663

[86] Wang Z, Liang L, Yin Z, Lin J. Improving chemical similarity ensemble approach in target prediction. J Cheminform. 2016;8:20. 27110288 10.1186/s13321-016-0130-x PMC4842302

[87] Mousavian Z, Khakabimamaghani S, Kavousi K, Masoudi-Nejad A. Drug-target interaction prediction from PSSM based evolutionary information. J Pharmacol Toxicol Methods. 2016;78:42–51. 26592807 10.1016/j.vascn.2015.11.002

[88] Chen H, Zhang Z. A semi-supervised method for drug-target interaction prediction with consistency in networks. PLoS One. 2013;8(5):e62975. 23667553 10.1371/journal.pone.0062975 PMC3646965

[89] Lan W, Wang J, Li M, Liu J, Li Y, Wu F-X, Predicting drug–target interaction using positive-unlabeled learning. Neurocomputing. 2016;206:50–7.

[90] Ezzat A, Wu M, Li X-L, Kwoh C-K. Drug-target interaction prediction via class imbalance-aware ensemble learning. BMC Bioinformatics. 2016;17(19):267–76. 28155697 10.1186/s12859-016-1377-y PMC5259867

[91] Fu G, Ding Y, Seal A, Chen B, Sun Y, Bolton E. Predicting drug target interactions using meta-path-based semantic network analysis. BMC Bioinformatics. 2016;17:160. 27071755 10.1186/s12859-016-1005-x PMC4830032

[92] Wilkinson MD, Dumontier M, Aalbersberg IJ, Appleton G, Axton M, Baak A, The FAIR guiding principles for scientific data management and stewardship. Sci Data. 2016;3:160018. 26978244 10.1038/sdata.2016.18 PMC4792175

