# Supplementary material for: Large-Scale Prediction of Drug-Target Interaction: a Data-Centric Review
Source: AAPS J. Author manuscript; Available in PMC 2024 May 16. (PMC11097213; doi:10.1208/s12248-017-0092-6)
Supplement: Supplementary Table S1, S2 and S3 [file NIHMS1989936-supplement-Supplementary_Table_S1__S2_and_S3.docx]

Large-scale prediction of drug-target interaction: a data-centric review

Tiejun Cheng, Ming Hao, Takako Takeda, Stephen H. Bryant, and Yanli Wang^*^

National Center for Biotechnology Information, National Library of Medicine, National Institutes of Health, Bethesda, MD, 20894, USA

Corresponding author: Yanli Wang ([ywang@ncbi.nlm.nih.gov](mailto:ywang@ncbi.nlm.nih.gov))

Table S1. Applications of drug-target interaction (DTI) prediction

| **Study** | **Year** | **Title** | **Drug info.** | **Target info.** | **DTI info. ^a^** | **Model** |
| --- | --- | --- | --- | --- | --- | --- |
| Takarabe et al. (1) | 2012 | [Drug target prediction using adverse event report systems: a pharmacogenomic approach](https://bioinformatics.oxfordjournals.org/content/28/18/i611.full) | side effects from FAERS, SIDER and JAPIC;  chemical similarity | genomic sequence similarity | KEGG DRUG (6,769 interactions among 2,423 drugs and 436 targets) | pairwise kernel regression |
| Sawada et al. (2) | 2014 | [Benchmarking a Wide Range of Chemical Descriptors for Drug-Target Interaction Prediction Using a Chemogenomic Approach](http://onlinelibrary.wiley.com/doi/10.1002/minf.201400066/full) | chemical similarity from 18 chemical descriptors (e.g. ECFP, FCFP, E-state, CDK, Klekota-Roth, MACCS, PubChem, Dragon, KCF-S, and graph kernels) | 4 descriptors of proteins (e.g., amino acid composition, domain profile, local sequence similarity, and string kernel) | KEGG, DrugBank, Matador,  ChEMBL, and PDSP Ki (107,671 interactions among 4,308  drugs and 2,689  targets) | pairwise kernel regression |
| Vilar et al. (3) | 2016 | [Computational Drug Target Screening through Protein Interaction Profiles](https://www.ncbi.nlm.nih.gov/pmc/articles/PMC5109486/) | Target interaction profile fingerprints (TIPFs) |  | ChEMBL (449,996 DTIs among 11,548 compounds and 2,498 targets) | similarity search |
| Kim et al. (4) | 2013 | [Predicting Drug-Target Interactions Using Drug-Drug Interactions](http://journals.plos.org/plosone/article?id=10.1371/journal.pone.0080129) | Drug-drug interactions |  | ChEMBL, SIDER, STITCH, and drugs.com (4,438 DTIs among 444 drugs and 835 target proteins) | support vector machine (SVM) and a kernel-based L1-norm regularized logistic regression (KL1LR) |
| Lo et al. (5) | 2015 | [Large-Scale Chemical Similarity Networks for Target Profiling of Compounds Identified in Cell-Based Chemical Screens](http://journals.plos.org/ploscompbiol/article?id=10.1371/journal.pcbi.1004153) | chemical similarity calculated by OBabel using FP2 fingerprint |  | ChEMBL, PubChem | Chemical Similarity Network Analysis Pulldown (CSNAP) |
| Gong et al. (6) | 2013 | [ChemMapper: a versatile web server for exploring pharmacology and chemical structure association based on molecular 3D similarity method](http://bioinformatics.oxfordjournals.org/content/29/14/1827.long) | molecular 3D similarity (SHAFTS) |  | ChEMBL, DrugBank, BindingDB, KEGG, and PDB | similarity search |
| AbdulHameed et al. (7) | 2012 | [Exploring Polypharmacology Using a ROCS-Based Target Fishing Approach](http://pubs.acs.org/doi/full/10.1021/ci2003544) | molecular 3D similarity |  | DrugBank (245 targets, 1,150 drugs) | similarity search |
| Cheng, et al. (8) | 2011 | [Identifying compound-target associations by combining bioactivity profile similarity search and public databases mining](http://pubs.acs.org/doi/full/10.1021/ci200192v) | Bioactivity profile from NCI-60 |  | DrugBank (28 drugs, 44 targets), TTD (33 drugs, 50 targets), ChEMBL (23 drugs, 67 targets), PubChem (215 drugs, 1,046 targets) | similarity search |
| Nickel et al. (9) | 2014 | [SuperPred: update on drug classification and target prediction](http://nar.oxfordjournals.org/content/42/W1/W26.long) | 2D chemical similarity using ECFP4 |  | SuperTarget,  ChEMBL, BindingDB (∼341,000 compounds, ∼1,800 targets and ∼665,000 compound–target interactions)  TTD (221 targets, 95,000 compounds and 174,000 compound–target interactions) | similarity search |
| Wang et al. (10) | 2013 | [Predicting drug-target interactions using restricted Boltzmann machines](http://bioinformatics.oxfordjournals.org/content/29/13/i126.full) |  |  | MATADOR (784 drugs, 2,431 protein targets and 13,064 DTIs) and STITCH (598 drugs, 671 protein targets and 3,296 DTIs) | restricted Boltzmann machine |
| Chen et al. (11) | 2012 | [Assessing drug target association using semantic linked data](http://journals.plos.org/ploscompbiol/article?id=10.1371/journal.pcbi.1002574) | ontology | ontology | DrugBank (5,607 DTIs), MATADOR (1,176 DTIs) | Semantic Link Association Prediction (SLAP) |
| Cao et al. (12) | 2015 | [In silico study of polypharmacology with protein-ligand interacting fingerprint](http://www.smartscitech.com/index.php/rci/article/viewFile/976/pdf_127) | binding site | 3D structure |  | similarity search |
| Cheng et al. (13) | 2013 | [Prediction of polypharmacological profiles of drugs by the integration of chemical, side effect, and therapeutic space](http://pubs.acs.org/doi/full/10.1021/ci400010x) | side effect  2D structure  ATC code |  | DrugBank, TTD (3195 DTIs from 621 drugs and 893 targets) | drug side effect similarity inference (DSESI) |
| Yang et al. (14) | 2014 | [Drug-target interaction prediction by integrating chemical, genomic, functional and pharmacological data](http://www.worldscientific.com/doi/abs/10.1142/9789814583220_0015) | side effect from SIDER, KEGG, JAPIC and AERS  chemical similarity | sequence similarity  biological function (GO) | KEGG (2,596 DTIs from 875 drugs and 249 proteins) | conditional random field (CRF) |
| Zhu et al. (15) | 2005 | [A probabilistic model for mining implicit ‘chemical compound–gene’ relations from literature](http://bioinformatics.oxfordjournals.org/content/21/suppl_2/ii245.full.pdf) | chemical structure |  | ChEBI | mixture aspect model (MAM) |
| Yu et al. (16) | 2012 | [A Systematic Prediction of Multiple Drug-Target Interactions from Chemical, Genomic, and Pharmacological Data](http://journals.plos.org/plosone/article?id=10.1371/journal.pone.0037608) | chemical descriptor calculated by Dragon | sequence descriptor calculated by PROFEAT | DrugBank (6,511 drugs , 3987 targets) | Random Forest (RF) and Support Vector Machine (SVM) |
| Nanni et al. (17) | 2014 | [A set of descriptors for identifying the protein–drug interaction in cellular networking](http://www.sciencedirect.com/science/article/pii/S0022519314003452) | FP2 calculated by OpenBabel | amino acid sequence (AAS)  position specific scoring matrix (PSSM)  substitution matrix representation (SMR)  wavelet (Wave) | three datasets from other studies (1,327 DTIs for ion channel; 620 DTIs for GPCR; 2,719 DTIs for enzyme) | ensemble of support vector machines (SVMs) |
| Gao et al. (18) | 2013 | [Prediction of Drugs Target Groups Based on ChEBI Ontology](https://www.hindawi.com/journals/bmri/2013/132724/) | ChEBI ontology |  | KEGG (876 drugs) | ontology similarity |
| Fakhraei  et al. (19) | 2013 | [Drug-Target Interaction Prediction for Drug Repurposing with Probabilistic Similarity Logic](http://linqs.cs.umd.edu/basilic/web/Publications/2013/fakhraei:biokdd13/FakhraeiBioKDD13.pdf) | chemical similarity (CDK)  ligand similarity (SEA)  gene expression (CMap)  side effect (SIDER)  annotation (ATC) | Sequence (Smith-Waterman score)  PPI-network  Gene Ontology | DrugBank (315 drugs, 250 targets and 1,306 interactions) | probabilistic similarity logic |
| Tao et al. (20) | 2015 | [Colorectal cancer drug target prediction using ontology-based inference and network analysis](http://database.oxfordjournals.org/content/2015/bav015.full) | ontology | ontology | PharmGKB, DrugBank, TTD | Ontology reasoning with network-assisted gene ranking |
| Sun et al. (21) | 2016 | [Side effect profile similarities shared between antidepressants and immune-modulators reveal potential novel targets for treating major depressive disorders](https://bmcpharmacoltoxicol.biomedcentral.com/articles/10.1186/s40360-016-0090-9) | side effects from SIDER |  | KEGG DRUG | similarity comparison |
| Hizukuri et al. (22) | 2015 | [Predicting target proteins for drug candidate compounds based on drug-induced gene expression data in a chemical structure-independent manner](http://bmcmedgenomics.biomedcentral.com/articles/10.1186/s12920-015-0158-1) | drug-induced gene expression from CMap  chemical similarity calculated by DragonX | sequence similarity | DrugBank, ChEMBL (4,870 DTIs from 756 compounds and 584 proteins) | pairwise kernel regression (PKR) |
| Wang et al. (23) | 2013 | [Drug target predictions based on heterogeneous graph inference](http://www.worldscientific.com/doi/pdf/10.1142/9789814447973_0006) | chemical similarity calculated by CDK | sequence similarity calculated by e Smith‐Waterman algorithm | DrugBank (2,098 DTIs from 1,409 drugs and 3,997 targets) | graph-based inference |
| Vilar et al. (24) | 2016 | [Leveraging 3D chemical similarity, target and phenotypic data in the identification of drug-protein and drug-adverse effect associations](http://jcheminf.springeropen.com/articles/10.1186/s13321-016-0147-1) | 3D chemical similarity |  | ChEMBL (22,838 DTIs from 1,526 drugs and 726 targets) | shape screening by Phase |
| Liu et al. (25) | 2015 | [Improving compound–protein interaction prediction by building up highly credible negative samples](https://bioinformatics.oxfordjournals.org/content/31/12/i221.full) | chemical similarity using PubChem fingerprint  side effects (SIDER) | sequence similarity calculated by normalized version of Smith-Waterman score  functional annotation semantic similarity (GO)  protein domain similarity (PFAM) | DrugBank, Matador, STITCH (2,290,630 DTIs from 367,142 compounds and 19,342 human proteins) | bipartite local model  Gaussian kernel profile  Bayesian matrix factorization |
| Tabei et al. (26) | 2013 | [Scalable prediction of compound-protein interactions using minwise hashing](http://bmcsystbiol.biomedcentral.com/articles/10.1186/1752-0509-7-S6-S3) | chemical similarity using PubChem fingerprint | domain fingerprint (PFAM) | STITCH (300,202 DTIs from 35,366 compounds and 6,111 proteins) | linear SVM |
| Jaeger et al. (27) | 2014 | [Causal Network Models for Predicting Compound Targets and Driving Pathways in Cancer](http://journals.sagepub.com/doi/full/10.1177/1087057114522690) | gene expression (CMap) |  | Metabase, STRING | causal graph-based analysis |
| Carrella et al. (28) | 2014 | [Mantra 2.0: an online collaborative resource for drug mode of action and repurposing by network analysis](http://bioinformatics.oxfordjournals.org/content/30/12/1787.full) | drug treated gene expression (CMap) |  |  | similarity comparison based on prototype ranked list |
| Yamanishi et al. (29) | 2014 | [DINIES: drug–target interaction network inference engine based on supervised analysis](http://nar.oxfordjournals.org/content/42/W1/W39.long) | chemical structures  drug side effects | amino acid sequences  and protein domains | KEGG | drug-target interaction network inference engine based on supervised analysis (DINIES) |
| Zheng et al. (30) | 2015 | [Large-scale Direct Targeting for Drug Repositioning and Discovery](http://www.nature.com/articles/srep11970) | chemical similarity calculated by CDK and Dragon |  | BindingDB (822,643 DTIs from 5,311 proteins and 490,282 ligands) | weighted ensemble similarity (WES) |
| Ravindranath et al. (31) | 2015 | [Connecting gene expression data from connectivity map and in silico target predictions for small molecule mechanism-of-action analysis](http://pubs.rsc.org/is/content/articlehtml/2015/mb/c4mb00328d) | chemical similarity (ECFP4)  gene expression (CMap) |  | ChEMBL (190,000 DTIs from 477 protein targets) | Laplacian-modified Nave Bayes classifier (NB) |
| Phatak et al. (32) | 2013 | [A novel multi-modal drug repurposing approach for identification of potent ACK1 inhibitors](http://www.worldscientific.com/doi/pdf/10.1142/9789814447973_0004) | chemical similarity calculated by Openbabel | sequence similarity calculated by Needleman-Wunsch algorithm | DrugBank, PDB, and UniProt | chemical-genomic similarity methods, combined with molecular graph theories |
| Perot et al. (33) | 2013 | [Insights into an Original Pocket-Ligand Pair Classification: A Promising Tool for Ligand Profile Prediction](http://journals.plos.org/plosone/article?id=10.1371/journal.pone.0063730) | binding site  ligand descriptor calculated by FAF-Drug2 |  | PDBbind (4,057 DTIs), Astex | multivariate analysis |
| Soufan et al. (34) | 2015 | [Mining Chemical Activity Status from High-Throughput Screening Assays](http://journals.plos.org/plosone/article?id=10.1371/journal.pone.0144426) | chemical fingerprints (RDKit, OpenBabel, PubChem) |  | PubChem BioAssay (500,000 DTIs) | DRAMOTE (minority oversampling technique) |
| Lusci et al. (35) | 2015 | [Accurate and efficient target prediction using a potency-sensitive influence-relevance voter](http://jcheminf.springeropen.com/articles/10.1186/s13321-015-0110-6) | chemical fingerprint (ECFP-like) |  | ChEMBL (490,760 DTIs from 3,236 proteins) | Potency-sensitive influence relevance voter (PS-IRV) |
| Alvarsson et al. (36) | 2014 | [Ligand-Based Target Prediction with Signature Fingerprints](http://pubs.acs.org/doi/full/10.1021/ci500361u) | six fingerprints (CountTc1, CountTc2, CDKExtended, FOYFI, ECFP, ECFI) |  | ChEMBL (284,186 DTIs from 99,790 compounds and 161 targets) | similarity search |
| Sugaya (37) | 2013 | [Training Based on Ligand Efficiency Improves Prediction of Bioactivities of Ligands and Drug Target Proteins in a Machine Learning Approach](http://pubs.acs.org/doi/full/10.1021/ci400240u) | three fingerprints (MACCS, MACCSF, and TGT) calculated by MOE  binding site descriptor | diAA descriptor based on amino acid sequence | ChEMBL (training set 5: 666,313 DTIs from 2,809 targets and 393,090 compounds) | SVM |
| Lounkine et al. (38) | 2012 | [Large-scale prediction and testing of drug activity on side-effect targets](http://www.nature.com/nature/journal/v486/n7403/full/nature11159.html) | chemical similarity (ECFP4 and Daylight) |  |  | chemical similarity ensemble approach (SEA) |
| Wang et al. (39) | 2016 | [Improving chemical similarity ensemble approach in target prediction](https://jcheminf.springeropen.com/articles/10.1186/s13321-016-0130-x) | 6 fingerprints (Morgan, atom paire, topological torsions, MACCS keys, 2D pharmacophore, and SHED) |  | ChEMBL | chemical similarity ensemble approach (SEA) |
| Wang et al. (40) | 2015 | [Predicting target-ligand interactions using protein ligand-binding site and ligand substructures](http://bmcsystbiol.biomedcentral.com/articles/10.1186/1752-0509-9-S1-S2) | binding site  PubChem fingerprint  functional groups by Check-mol |  | sc-PDB (6,830 DTIs from 2,710 ligands and 836 targets) | fragment interaction model (FIM) |
| Zhou et al. (41) | 2015 | [Comprehensive prediction of drug-protein interactions and side effects for the human proteome](http://www.nature.com/articles/srep11090) | binding site |  | human proteome | [FINDSITE^comb^](http://pubs.acs.org/doi/abs/10.1021/ci300510n) ligand homology approach |
| Kuang et al. (42) | 2015 | [An eigenvalue transformation technique for predicting drug-target interaction](http://www.nature.com/articles/srep13867) | ATC code  chemical similarity calculated by SIMCOMP | sequence similarity calculated by normalized Smith-Waterman score | DrugBank (3,681 DTIs from 786 drugs and 809 targets) | RLS and SLP with  an eigenvalue transformation technique |
| Meslamani et al. (43) | 2011 | [Enhancing the accuracy of chemogenomic models with a three-dimensional binding site kernel](http://pubs.acs.org/doi/full/10.1021/ci200166t) | binding site  chemical similarity (ECFP4) | three descriptors (UniProt name, SPECTRUM sequence-based, 3-D structural descriptor) | sc-PDB (2,882 DTIs from 581 targets and 2,605 ligands) | SVM |
| Shaikh et al. (44) | 2016 | [An improved approach for predicting drug–target interaction: proteochemometrics to molecular docking](http://pubs.rsc.org/en/content/articlehtml/2016/mb/c5mb00650c) | chemical descriptor (Morgan fingerprint by RDKit) | protein descriptor calculated by PROFEAT | sc-PDB (3,063 DTIs from 1,473 proteins and 2,040 targets) | SVM, RF, Naive Bayes, k-NN |
| Xiao et al. (45) | 2015 | [iDrug-Target: predicting the interactions between drug compounds and target proteins in cellular networking via benchmark dataset optimization approach](http://www.tandfonline.com/doi/full/10.1080/07391102.2014.998710) | 2D molecular fingerprint | pseudo amino acid composition | 620 DTIs for 620 GPCRs; 1,372 DTIs for ion channels; 2,719 DTIs for enzymes; 86 DTIs for nuclear receptors | SVM |
| Martínez-Jiménez et al. (46) | 2015 | [Ligand-target prediction by structural network biology using nAnnoLyze](http://journals.plos.org/ploscompbiol/article?id=10.1371/journal.pcbi.1004157) | weighted quantitative estimate of drug-likeness (wQED)  binding site | PDB  human 3D models (ModBase) | PDB | nAnnoLyze |
| Hu et al. (47) | 2016 | [Predicting Drug-Target Interactions Based on Small Positive Samples](https://www.ncbi.nlm.nih.gov/pubmed/27829343) |  |  |  | one-class classification algorithm |
| Cobanoglu et al. (48) | 2013 | [Predicting Drug−Target Interactions Using Probabilistic Matrix Factorization](http://pubs.acs.org/doi/full/10.1021/ci400219z) |  |  | DrugBank (4,731 DTIs from 1,413 drugs and 1,050 targets) | probabilistic matrix factorization (PMF) |
| Ezzat et al. (49) | 2016 | [Drug-target interaction prediction via class imbalance-aware ensemble learning](https://bmcbioinformatics.biomedcentral.com/articles/10.1186/s12859-016-1377-y) | chemical descriptors calculated by *Rcpi*  package | target feature calculated by PROFEAT | DrugBank (12,674 DTIs from 5,877 drugs and 3,348 targets) | ensemble learning method |
| Liu et al. (50) | 2014 | [In Silico target fishing: addressing a “Big Data” problem by ligand-based similarity rankings with data fusion](https://jcheminf.springeropen.com/articles/10.1186/1758-2946-6-33) | chemical similarity (ECFP4) |  | BindingDB (246,053 DTIs among 179,807 compounds and 533 targets), DrugBank (7,917 DTIs among 711 drugs and 455 targets), and TTD (1,084 DTIs among 476 drugs and 255 targets) | similarity search |
| Fu et al. (51) | 2016 | [Predicting drug target interactions using meta-path-based semantic network analysis](https://bmcbioinformatics.biomedcentral.com/articles/10.1186/s12859-016-1005-x) | semantic annotation | semantic annotation | DrugBank (5,387 DTIs), PubChem (180,000 DTIs) | Semantic Link Association Prediction (SLAP) |
| Cheng et al. (52) | 2012 | [Prediction of Chemical-Protein Interactions Network with Weighted Network-Based Inference Method](http://journals.plos.org/plosone/article?id=10.1371/journal.pone.0041064) | chemical similarity calculated by OpenBabel using MACCS keys | sequence similarity calculated by Smith-Waterman score | ChEMBL (17,111 CPIs from 4,741 compounds and 97 GPCRs; 13,648 CPIs from 2,827 compounds and 206 kinases) | edge-weighted NBI |
| Alaimo et al. (53) | 2015 | [DT-Web: a web-based application for drug-target interaction and drug combination prediction through domain-tuned network-based inference](http://bmcsystbiol.biomedcentral.com/articles/10.1186/1752-0509-9-S3-S4) |  | biological function (GO) | DrugBank | DT-Hybrid |
| Flores et al. (54) | 2015 | [Exploiting Semantics to Predict Potential Novel Links from Dense Subgraphs](http://ceur-ws.org/Vol-1378/AMW_2015_paper_26.pdf) |  |  | dataset from other study based on benchmark dataset (5,000 DTIs from 900 drugs and 1,000 targets)  STITCH and KEGG | Edge-Similarity Densest Subgraph (esDSG) |
| Zhang, et al. (55) | 2016 | [DrugRPE: Random projection ensemble approach to drug-target interaction prediction](http://www.sciencedirect.com/science/article/pii/S0925231216312590) | descriptor calculated by PaDEL | amino acid properties and sequence profile | dataset from other study that is derived from benchmark dataset | random projection ensemble (REPTree) |
| Palma et al. (56) | 2014 | [Drug-Target Interaction Prediction Using Semantic Similarity and Edge Partitioning](http://link.springer.com/chapter/10.1007/978-3-319-11964-9_9) | disease terms |  | benchmark dataset based (5,000 DTIs from 900 drugs and 1,000 targets) | unsupervised semantics based edge partitioning approach (semEP) |
| Öztürk al. (57) | 2016 | [A comparative study of SMILES-based compound similarity functions for drug-target interaction prediction](https://bmcbioinformatics.biomedcentral.com/articles/10.1186/s12859-016-0977-x) | SMILES-based similarity | sequence similarity (Smith-Waterman score) | benchmark dataset | Weighted Nearest Neighbor algorithm (WNN-GIP) |
| Ba-alawi et al. (58) | 2016 | [DASPfind: new efficient method to predict drug–target interactions](https://www.ncbi.nlm.nih.gov/pmc/articles/PMC4793623/) | chemical similarity calculated by SIMCOMP | sequence similarity calculated by normalized Smith-Waterman algorithm | benchmark dataset | graph traversal (DASPfind) |
| Chen et al. (59) | 2012 | [Drug–target interaction prediction by random walk on the heterogeneous network](http://pubs.rsc.org/en/content/articlehtml/2012/mb/c2mb00002d) | chemical similarity calculated by SIMCOMP | sequence similarity calculated by normalized Smith-Waterman score | benchmark dataset | Network-based Random Walk with Restart on the Heterogeneous network (NRWRH) |
| Alaimo et al. (60) | 2013 | [Drug–target interaction prediction through domain-tuned network-based inference](https://bioinformatics.oxfordjournals.org/content/29/16/2004.full) | chemical similarity calculated by SIMCOMP | Smith-Waterman sequence similarity | benchmark dataset based | domain tuned-hybrid (DT-Hybrid) |
| Gönen (61) | 2012 | [Predicting drug–target interactions from chemical and genomic kernels using Bayesian matrix factorization](http://bioinformatics.oxfordjournals.org/content/28/18/2304.full) | chemical similarity calculated by SIMCOMP | sequence similarity calculated by normalized Smith-Waterman score | benchmark dataset | kernelized Bayesian matrix factorization with twin kernels (KBMF2K) |
| Shi et al. (62) | 2015 | [Predicting drug–target interaction for new drugs using enhanced similarity measures and super-target clustering](http://www.sciencedirect.com/science/article/pii/S1046202315001905) | chemical similarity  ATC code | sequence similarity calculated by Smith-Waterman alignment score  function categories (FC)-based semantic similarity | benchmark dataset |  |
| Cheng et al. (63) | 2012 | [Prediction of Drug-Target Interactions and Drug Repositioning via Network-Based Inference](http://journals.plos.org/ploscompbiol/article?id=10.1371/journal.pcbi.1002503) | chemical similarity calculated by SIMCOMP | sequence similarity by  normalized version of Smith-Waterman scores | benchmark dataset based | drug-based similarity inference (DBSI)  target-based similarity inference (TBSI)  network-based inference (NBI) |
| Geethanjali et al. (64) | 2016 | [Generating Drug-Gene Association for Vibrio Cholerae using Ontological Profile Similarity](http://www.indjst.org/index.php/indjst/article/view/99620/72982) | ontology |  |  | Pointwise Mutual Information (PMI) |
| Lan et al. (65) | 2015 | [Predicting drug-target interaction based on sequence and structure information](http://www.sciencedirect.com/science/article/pii/S2405896315027160) | structure similarity calculated by SIMCOMP | sequence similarity calculated by Smith-Waterman score | benchmark dataset | Weighted SVM |
| Cao et al. (66) | 2012 | [Large-scale prediction of drug–target interactions using protein sequences and drug topological structures](http://www.sciencedirect.com/science/article/pii/S0003267012013487) | chemical similarity calculated by OpenBabel using MACCS fingerprint | 167 protein descriptors and 20 amino acid composition descriptors calculated by ProPy | benchmark dataset | SVM |
| Chen et al. (67) | 2013 | [A Semi-Supervised Method for Drug-Target Interaction Prediction with Consistency in Networks](http://journals.plos.org/plosone/article?id=10.1371/journal.pone.0062975) | chemical similarity calculated by SIMCOMP | sequence similarity calculated by normalized version of Smith-Waterman score | benchmark dataset | Network-Consistency-based Prediction Method (NetCBP) |
| Shi et al. (68) | 2016 | [Predicting existing targets for new drugs base on strategies for missing interactions](https://bmcbioinformatics.biomedcentral.com/articles/10.1186/s12859-016-1118-2) | chemical similarity  ATC code | sequence similarity calculated by Smith-Waterman alignment  functional categories (HUGO annotation) | benchmark dataset | RLSm_comb, RLSm_super, RLSm_spy |
| Laarhoven et al. (69) | 2013 | [Predicting Drug-Target Interactions for New Drug Compounds Using a Weighted Nearest Neighbor Profile](http://journals.plos.org/plosone/article?id=10.1371/journal.pone.0066952) | chemical similarity calculated by SIMCOMP | sequence similarity calculated by normalized Smith-Waterman score | benchmark dataset | WNN-GIP |
| Mei et al. (70) | 2013 | [Drug–target interaction prediction by learning from local information and neighbors](http://bioinformatics.oxfordjournals.org/content/29/2/238.full) | chemical similarity calculated by SIMCOMP | sequence similarity calculated by normalized Smith-Waterman score | benchmark dataset | BLM–NII |
| Zhao et al. (71) | 2015 | [A Label Extended Semi-supervised Learning Method for Drug-target Interaction Prediction](http://www.atlantis-press.com/php/download_paper.php?id=20307) | chemical similarity calculated by SIMCOMP | sequence similarity calculated by normalized Smith-Waterman score | benchmark dataset | label extended semi-supervised learning method (LESSL) |
| Lan et al. (72) | 2016 | [Predicting drug–target interaction using positive-unlabeled learning](http://www.sciencedirect.com/science/article/pii/S0925231216304337) | chemical similarity calculated by SIMCOMP | sequence similarity calculated by normalized Smith-Waterman score | benchmark dataset | Positive-unlabeled learning |
| Shi et al. (73) | 2013 | [Protein-chemical interaction prediction via kernelized sparse learning SVM](http://www.worldscientific.com/doi/abs/10.1142/9789814447973_0005) | chemical similarity by ECFP4 | sequence similarity based on local binding region | benchmark dataset | L1-norm SVM |
| Peng et al. (74) | 2015 | [Predicting Drug–Target Interactions With Multi-Information Fusion](http://www.cs.newpaltz.edu/~lik/publications/Lihong-Peng-IEEE-JBHI-2016.pdf) | chemical similarity calculated by SIMCOMP | sequence similarity calculated by normalized Smith-Waterman score | benchmark dataset | semi-supervised based learning framework (NormMulInf) |
| Wang et al. (75) | 2013 | [Computational Study of Drugs by Integrating Omics Data with Kernel Methods](http://onlinelibrary.wiley.com/doi/10.1002/minf.201300090/full) | chemical similarity (SIMCOMP)  side effects (JAPIC, SIDER)  ATC code  activity data | sequence similarity calculated by normalized Smith-Waterman score | benchmark dataset | SVM-based algorithm |
| Niu (76) | 2014 | [Supervised prediction of drug-target interactions by ensemble learning](http://www.jocpr.com/articles/supervised-prediction-of-drugtarget-interactions-by-ensemble-learning.pdf) | chemical similarity calculated by SIMCOMP | sequence similarity using Smith-Waterman scores | benchmark dataset | Ensemble learning with random forest as the classification engine |
| van Laarhoven et al. (77) | 2011 | [Gaussian interaction profile kernels for predicting drug-target interaction](http://bioinformatics.oxfordjournals.org/content/27/21/3036.full) |  |  | benchmark dataset | Gaussian Interaction Profile (GIP) |
| Hao et al. (78) | 2016 | [Improved prediction of drug-target interactions using regularized least squares integrating with kernel fusion technique](http://www.sciencedirect.com/science/article/pii/S0003267016300630) | chemical similarity calculated by SIMCOMP | sequence similarity using Smith-Waterman scores | benchmark dataset | regularized least squares integrating with nonlinear kernel fusion (RLS-KF) |
| Nascimento et al. (79) | 2016 | [A multiple kernel learning algorithm for drug-target interaction prediction](https://bmcbioinformatics.biomedcentral.com/articles/10.1186/s12859-016-0890-3) | chemical similarity calculated by SIMCOMP  side effects (AERS, SIDER) | Mismatch and Spectrum calculated by KeBABS  Geno Ontology (GO)  protein-protein network (PPI) | benchmark dataset | KronRLS-MKL |
| Liu et al. (80) | 2016 | [Neighborhood regularized logistic matrix factorization for drug-target interaction prediction](http://journals.plos.org/ploscompbiol/article?id=10.1371/journal.pcbi.1004760) | chemical similarity calculated by SIMCOMP | sequence similarity using Smith-Waterman scores | benchmark dataset | neighborhood regularized logistic matrix factorization (NRLMF) |
| Mousavian et al. (81) | 2015 | [Drug-target interaction prediction from PSSM based evolutionary information](http://www.sciencedirect.com/science/article/pii/S1056871915002944) |  | position Specific Scoring Matrix (PSSM) | benchmark dataset | Bigram-PSSM |
| Cao et al. (82) | 2014 | [Computational prediction of drug-target interactions using chemical, biological, and network features](http://onlinelibrary.wiley.com/doi/10.1002/minf.201400009/full) | chemical similarity calculated by OpenBabel using MACCS keys | amino acid composition, composition (C), transition (T) and distribution (D) | benchmark dataset | Random Forest (RF) |
| Yan et al. (83) | 2016 | [Prediction of drug-target interaction by label propagation with mutual interaction information derived from heterogeneous network](http://pubs.rsc.org/en/content/articlehtml/2016/mb/c5mb00615e) | chemical similarity calculated by SIMCOMP | sequence similarity using Smith-Waterman scores | benchmark dataset  Kd (1,527 DTIs from 68 drugs and 442 targets) and Ki (3,200 DTIs from 1,421 drugs and 156 targets) data sets from other study | label propagation with mutual interaction information derived from heterogeneous networks (LPMIHN) |
| Koohi et al. (84) | 2013 | [Prediction of drug-target interactions using popular collaborative filtering methods](http://ieeexplore.ieee.org/stamp/stamp.jsp?arnumber=6735931) |  |  | benchmark dataset | collaborative filtering (CF) |
| Zheng et al. (85) | 2013 | [Collaborative matrix factorization with multiple similarities for predicting drug-target interactions](http://www.bic.kyoto-u.ac.jp/pathway/Files/kdd13.pdf) | chemical structure similarity  ATC similarity | sequence similarity by normalized Smith-Waterman score  Gene Ontology similarity  PPI network similairty | benchmark dataset | Multiple Similarities Collaborative Matrix Factorization (MSCMF) |
| Ezzat et al. (86) | 2016 | [Drug-target interaction prediction with graph regularized matrix factorization](http://ieeexplore.ieee.org/stamp/stamp.jsp?arnumber=7407341) | chemical similarity calculated by SIMCOMP | sequence similarity using Smith-Waterman scores | benchmark dataset | Graph Regularized Matrix Factorization (GRMF) |

^a^ The benchmark dataset was presented in Yamanishi et al.(2008) (87), which contains four subsets for enzyme (445 drugs, 664 targets, and 2,926 DTIs), ion channels (210 drugs, 204 targets, and 1,476 DTIs), GPCR (223 drugs, 95 targets, and 635 DTIs), and nuclear receptor (54 drugs, 26 targets, and 90 DTIs), respectively. The dataset can be downloaded at: <http://web.kuicr.kyoto-u.ac.jp/supp/yoshi/drugtarget/>.

Table S2. Software and web servers for feature/descriptor and similarity calculation for drugs and targets

| **Software** | **Link** | **Ref.** |
| --- | --- | --- |
| SIMCOMP | <http://www.genome.jp/tools/simcomp/> | (96) |
| DRAGON | <http://www.talete.mi.it/products/dragon_molecular_descriptors.htm> | (97) |
| PaDEL | <http://www.yapcwsoft.com/dd/padeldescriptor/> | (98) |
| TOMOCOMD-CARDD | <http://tomocomd.com/> | (99) |
| e-DRAGON | <http://www.vcclab.org/lab/edragon/> | (100) |
| MOLD2 | <http://www.fda.gov/ScienceResearch/BioinformaticsTools/Mold2/> | (101) |
| CDK | <https://sourceforge.net/projects/cdk/> | (102) |
| OpenBabel | <http://openbabel.org/> | (103) |
| PROFEAT | <http://bidd2.nus.edu.sg/cgi-bin/profeat2016/main.cgi> | (104) |
| FAF-Drug3 | <http://fafdrugs3.mti.univ-paris-diderot.fr/> | (105) |
| QSARINS | <http://www.qsar.it/> | (106) |

Table S3**.** Online tools for predicting drug-target interaction

| **Resource** | **Description** | **Method** | **Link** | **Ref.** |
| --- | --- | --- | --- | --- |
| SuperPred | A prediction webserver for ATC code and target prediction of compounds | a combination of 2D, 3D, and fragment similarity values | <http://prediction.charite.de/> | (9) |
| SwissTargetPrediction | A web server to predict the targets of a small molecule | a combination of 2D and 3D similarity values | <http://www.swisstargetprediction.ch/> | (88) |
| DINIES | A drug-target interaction network inference engine based on drug data (derived from various data such as chemical structures, previously reported side-effect information, etc) and omics-scale protein data (derived from various data such as sequence similarity, domain composition, expression profile, etc) | network-based inference | <http://www.genome.jp/tools/dinies/> | (89) |
| TargetHunter | A web server designed and constructed to identify possible targets of small molecules by searching the available bioactive compound-target pairs reported from literature using the query structure | fingerprint based | <http://www.cbligand.org/TargetHunter/> | (90) |
| iDrug-Target | A package of web-services for predicting drug-target interaction | fingerprint based, machine learning based | <http://www.jci-bioinfo.cn/iDrug-Target/> | (45) |
| SEA | A search tool based on the similarity ensemble approach (SEA) for relating proteins based on the set-wise chemical similarity among their ligands. | fingerprint based | <http://sea.bkslab.org/> | (91) |
| DT-Web | A web-based interface for computing recommendations for each drug by combining domain-specific knowledge expressing drugs and targets similarity. | DTI and Drug combination | <https://alpha.dmi.unict.it/dtweb/> | (53) |
| ChemMapper | A web server for exploring pharmacology and chemical structure association based on molecular 3D similarity method | 3D similarity based | <http://lilab.ecust.edu.cn/chemmapper/> | (6) |
| HitPick | A web server for hit identification and target prediction of chemical screenings | combines 2D fingerprints and a machine learning method | <http://mips.helmholtz-muenchen.de/proj/hitpick> | (92) |
| SPiDER | A tool for the macromolecular targets of de novo-designed chemical entities through self-organizing map consensus | Self-organizing map-based prediction | <http://modlab-cadd.ethz.ch/software/spider/> | (93) |
| TargetNet | A web service for predicting potential drug-target interaction profiling via multi-target SAR models | SAR/fingerprint-based | <http://targetnet.scbdd.com> | (94) |
| nAnnoLyze | A online method that given a compound predicts its protein targets using a prebuilt network based on structural similarities | network-based | <http://www.marciuslab.org/services/nAnnoLyze> | (46) |
| CSNAP | A web server for compound target identification based on large-scale chemical similarity networks | similarity-based, network-based | <http://services.mbi.ucla.edu/CSNAP/> | (95) |

**References**

1. Takarabe M, Kotera M, Nishimura Y, Goto S, Yamanishi Y. Drug target prediction using adverse event report systems: a pharmacogenomic approach. Bioinformatics. 2012;28(18):i611-i8.

2. Sawada R, Kotera M, Yamanishi Y. Benchmarking a wide range of chemical descriptors for drug-target interaction prediction using a chemogenomic approach. Mol Inform. 2014;33(11-12):719-31.

3. Vilar S, Quezada E, Uriarte E, Costanzi S, Borges F, Vina D, et al. Computational Drug Target Screening through Protein Interaction Profiles. Sci Rep. 2016;6:36969.

4. Kim S, Jin D, Lee H. Predicting drug-target interactions using drug-drug interactions. PLoS One. 2013;8(11):e80129.

5. Lo YC, Senese S, Li CM, Hu Q, Huang Y, Damoiseaux R, et al. Large-scale chemical similarity networks for target profiling of compounds identified in cell-based chemical screens. PLoS Comput Biol. 2015;11(3):e1004153.

6. Gong J, Cai C, Liu X, Ku X, Jiang H, Gao D, et al. ChemMapper: a versatile web server for exploring pharmacology and chemical structure association based on molecular 3D similarity method. Bioinformatics. 2013;29(14):1827-9.

7. AbdulHameed MD, Chaudhury S, Singh N, Sun H, Wallqvist A, Tawa GJ. Exploring polypharmacology using a ROCS-based target fishing approach. J Chem Inf Model. 2012;52(2):492-505.

8. Cheng T, Li Q, Wang Y, Bryant SH. Identifying compound-target associations by combining bioactivity profile similarity search and public databases mining. J Chem Inf Model. 2011;51(9):2440-8.

9. Nickel J, Gohlke BO, Erehman J, Banerjee P, Rong WW, Goede A, et al. SuperPred: update on drug classification and target prediction. Nucleic Acids Res. 2014;42(Web Server issue):W26-31.

10. Wang Y, Zeng J. Predicting drug-target interactions using restricted Boltzmann machines. Bioinformatics. 2013;29(13):i126-34.

11. Chen B, Ding Y, Wild DJ. Assessing drug target association using semantic linked data. PLoS Comput Biol. 2012;8(7):e1002574.

12. Cao R, Wang Y. In silico study of polypharmacology with ligand-based interaction fingerprint. Receptors Clin Investig. 2015;2(4):e976.

13. Cheng F, Li W, Wu Z, Wang X, Zhang C, Li J, et al. Prediction of polypharmacological profiles of drugs by the integration of chemical, side effect, and therapeutic space. J Chem Inf Model. 2013;53(4):753-62.

14. Yang F, Xu J, Zeng J. Drug-target interaction prediction by integrating chemical, genomic, functional and pharmacological data. Pac Symp Biocomput. 2014:148-59.

15. Zhu S, Okuno Y, Tsujimoto G, Mamitsuka H. A probabilistic model for mining implicit 'chemical compound-gene' relations from literature. Bioinformatics. 2005;21 Suppl 2:ii245-51.

16. Yu H, Chen J, Xu X, Li Y, Zhao H, Fang Y, et al. A systematic prediction of multiple drug-target interactions from chemical, genomic, and pharmacological data. PLoS One. 2012;7(5):e37608.

17. Nanni L, Lumini A, Brahnam S. A set of descriptors for identifying the protein-drug interaction in cellular networking. J Theor Biol. 2014;359:120-8.

18. Gao YF, Chen L, Huang GH, Zhang T, Feng KY, Li HP, et al. Prediction of drugs target groups based on ChEBI ontology. Biomed Res Int. 2013;2013:132724.

19. Fakhraei S, Raschid L, Getoor L, editors. Drug-target interaction prediction for drug repurposing with probabilistic similarity logic. Proceedings of the 12th International Workshop on Data Mining in Bioinformatics; 2013: ACM.

20. Tao C, Sun J, Zheng WJ, Chen J, Xu H. Colorectal cancer drug target prediction using ontology-based inference and network analysis. Database. 2015;2015:bav015.

21. Sun Y, Narayan VA, Wittenberg GM. Side effect profile similarities shared between antidepressants and immune-modulators reveal potential novel targets for treating major depressive disorders. BMC Pharmacol Toxicol. 2016;17(1):47.

22. Hizukuri Y, Sawada R, Yamanishi Y. Predicting target proteins for drug candidate compounds based on drug-induced gene expression data in a chemical structure-independent manner. BMC Med Genomics. 2015;8:82.

23. Wang W, Yang S, Li J. Drug target predictions based on heterogeneous graph inference. Pac Symp Biocomput. 2013:53-64.

24. Vilar S, Hripcsak G. Leveraging 3D chemical similarity, target and phenotypic data in the identification of drug-protein and drug-adverse effect associations. J Cheminform. 2016;8:35.

25. Liu H, Sun J, Guan J, Zheng J, Zhou S. Improving compound-protein interaction prediction by building up highly credible negative samples. Bioinformatics. 2015;31(12):i221-9.

26. Tabei Y, Yamanishi Y. Scalable prediction of compound-protein interactions using minwise hashing. BMC Syst Biol. 2013;7 Suppl 6:S3.

27. Jaeger S, Min J, Nigsch F, Camargo M, Hutz J, Cornett A, et al. Causal Network Models for Predicting Compound Targets and Driving Pathways in Cancer. J Biomol Screen. 2014;19(5):791-802.

28. Carrella D, Napolitano F, Rispoli R, Miglietta M, Carissimo A, Cutillo L, et al. Mantra 2.0: an online collaborative resource for drug mode of action and repurposing by network analysis. Bioinformatics. 2014;30(12):1787-8.

29. Yamanishi Y, Kotera M, Moriya Y, Sawada R, Kanehisa M, Goto S. DINIES: drug-target interaction network inference engine based on supervised analysis. Nucleic Acids Res. 2014;42(Web Server issue):W39-45.

30. Zheng C, Guo Z, Huang C, Wu Z, Li Y, Chen X, et al. Large-scale direct targeting for drug repositioning and discovery. Sci Rep. 2015;5:11970.

31. Ravindranath AC, Perualila-Tan N, Kasim A, Drakakis G, Liggi S, Brewerton SC, et al. Connecting gene expression data from connectivity map and in silico target predictions for small molecule mechanism-of-action analysis. Mol BioSyst. 2015;11(1):86-96.

32. Phatak SS, Zhang S. A novel multi-modal drug repurposing approach for identification of potent ACK1 inhibitors. Pac Symp Biocomput. 2013:29-40.

33. Perot S, Regad L, Reynes C, Sperandio O, Miteva MA, Villoutreix BO, et al. Insights into an original pocket-ligand pair classification: a promising tool for ligand profile prediction. PLoS One. 2013;8(6):e63730.

34. Soufan O, Ba-alawi W, Afeef M, Essack M, Rodionov V, Kalnis P, et al. Mining Chemical Activity Status from High-Throughput Screening Assays. PLoS One. 2015;10(12):e0144426.

35. Lusci A, Browning M, Fooshee D, Swamidass J, Baldi P. Accurate and efficient target prediction using a potency-sensitive influence-relevance voter. J Cheminform. 2015;7:63.

36. Alvarsson J, Eklund M, Engkvist O, Spjuth O, Carlsson L, Wikberg JE, et al. Ligand-based target prediction with signature fingerprints. J Chem Inf Model. 2014;54(10):2647-53.

37. Sugaya N. Training based on ligand efficiency improves prediction of bioactivities of ligands and drug target proteins in a machine learning approach. J Chem Inf Model. 2013;53(10):2525-37.

38. Lounkine E, Keiser MJ, Whitebread S, Mikhailov D, Hamon J, Jenkins JL, et al. Large-scale prediction and testing of drug activity on side-effect targets. Nature. 2012;486(7403):361-7.

39. Wang Z, Liang L, Yin Z, Lin J. Improving chemical similarity ensemble approach in target prediction. J Cheminform. 2016;8:20.

40. Wang C, Liu J, Luo F, Deng Z, Hu QN. Predicting target-ligand interactions using protein ligand-binding site and ligand substructures. BMC Syst Biol. 2015;9 Suppl 1:S2.

41. Zhou H, Gao M, Skolnick J. Comprehensive prediction of drug-protein interactions and side effects for the human proteome. Sci Rep. 2015;5:11090.

42. Kuang Q, Xu X, Li R, Dong Y, Li Y, Huang Z, et al. An eigenvalue transformation technique for predicting drug-target interaction. Sci Rep. 2015;5:13867.

43. Meslamani J, Rognan D. Enhancing the accuracy of chemogenomic models with a three-dimensional binding site kernel. J Chem Inf Model. 2011;51(7):1593-603.

44. Shaikh N, Sharma M, Garg P. An improved approach for predicting drug-target interaction: proteochemometrics to molecular docking. Mol BioSyst. 2016;12(3):1006-14.

45. Xiao X, Min JL, Lin WZ, Liu Z, Cheng X, Chou KC. iDrug-Target: predicting the interactions between drug compounds and target proteins in cellular networking via benchmark dataset optimization approach. J Biomol Struct Dyn. 2015;33(10):2221-33.

46. Martinez-Jimenez F, Marti-Renom MA. Ligand-target prediction by structural network biology using nAnnoLyze. PLoS Comput Biol. 2015;11(3):e1004157.

47. Hu P, Chan KC, Hu Y. Predicting Drug-Target Interactions Based on Small Positive Samples. Curr Protein Pept Sci. 2016.

48. Cobanoglu MC, Liu C, Hu F, Oltvai ZN, Bahar I. Predicting drug-target interactions using probabilistic matrix factorization. J Chem Inf Model. 2013;53(12):3399-409.

49. Ezzat A, Wu M, Li X-L, Kwoh C-K. Drug-target interaction prediction via class imbalance-aware ensemble learning. BMC Bioinformatics. 2016;17(19):267-76.

50. Liu X, Xu Y, Li S, Wang Y, Peng J, Luo C, et al. In Silico target fishing: addressing a "Big Data" problem by ligand-based similarity rankings with data fusion. J Cheminform. 2014;6:33.

51. Fu G, Ding Y, Seal A, Chen B, Sun Y, Bolton E. Predicting drug target interactions using meta-path-based semantic network analysis. BMC Bioinformatics. 2016;17:160.

52. Cheng F, Zhou Y, Li W, Liu G, Tang Y. Prediction of chemical-protein interactions network with weighted network-based inference method. PLoS One. 2012;7(7):e41064.

53. Alaimo S, Bonnici V, Cancemi D, Ferro A, Giugno R, Pulvirenti A. DT-Web: a web-based application for drug-target interaction and drug combination prediction through domain-tuned network-based inference. BMC Syst Biol. 2015;9 Suppl 3:S4.

54. Flores A, Vidal M-E, Palma G, editors. Exploiting Semantics to Predict Potential Novel Links from Dense Subgraphs. Alberto Mendelzon International Workshop on Foundations of Data Management; 2015.

55. Zhang J, Zhu M, Chen P, Wang B. DrugRPE: Random projection ensemble approach to drug-target interaction prediction. Neurocomputing. 2016.

56. Palma G, Vidal M-E, Raschid L, editors. Drug-target interaction prediction using semantic similarity and edge partitioning. International Semantic Web Conference; 2014: Springer.

57. Ozturk H, Ozkirimli E, Ozgur A. A comparative study of SMILES-based compound similarity functions for drug-target interaction prediction. BMC Bioinformatics. 2016;17:128.

58. Ba-Alawi W, Soufan O, Essack M, Kalnis P, Bajic VB. DASPfind: new efficient method to predict drug-target interactions. J Cheminform. 2016;8:15.

59. Chen X, Liu MX, Yan GY. Drug-target interaction prediction by random walk on the heterogeneous network. Mol BioSyst. 2012;8(7):1970-8.

60. Alaimo S, Pulvirenti A, Giugno R, Ferro A. Drug-target interaction prediction through domain-tuned network-based inference. Bioinformatics. 2013;29(16):2004-8.

61. Gönen M. Predicting drug-target interactions from chemical and genomic kernels using Bayesian matrix factorization. Bioinformatics. 2012;28(18):2304-10.

62. Shi J-Y, Yiu S-M, Li Y, Leung HCM, Chin FYL. Predicting drug–target interaction for new drugs using enhanced similarity measures and super-target clustering. Methods. 2015;83:98-104.

63. Cheng F, Liu C, Jiang J, Lu W, Li W, Liu G, et al. Prediction of drug-target interactions and drug repositioning via network-based inference. PLoS Comput Biol. 2012;8(5):e1002503.

64. Geethanjali C, Bhanumathi S. Generating drug-gene association for Vibrio cholerae using ontological profile similarity. Indian J Sci Technol. 2016;9(33):99620.

65. Lan W, Wang J, Li M, Wu F-X, Pan Y. Predicting drug-target interaction based on sequence and structure information. IFAC-PapersOnLine. 2015;48(28):12-6.

66. Cao DS, Liu S, Xu QS, Lu HM, Huang JH, Hu QN, et al. Large-scale prediction of drug-target interactions using protein sequences and drug topological structures. Anal Chim Acta. 2012;752:1-10.

67. Chen H, Zhang Z. A semi-supervised method for drug-target interaction prediction with consistency in networks. PLoS One. 2013;8(5):e62975.

68. Shi JY, Li JX, Lu HM. Predicting existing targets for new drugs base on strategies for missing interactions. BMC Bioinformatics. 2016;17 Suppl 8:282.

69. van Laarhoven T, Marchiori E. Predicting drug-target interactions for new drug compounds using a weighted nearest neighbor Profile. PLoS One. 2013;8(6):e66952.

70. Mei JP, Kwoh CK, Yang P, Li XL, Zheng J. Drug-target interaction prediction by learning from local information and neighbors. Bioinformatics. 2013;29(2):238-45.

71. Zhao J, Cao Z. A Label Extended Semi-supervised Learning Method for Drug-target Interaction Prediction. AMCCE. 2015;13:21.

72. Lan W, Wang J, Li M, Liu J, Li Y, Wu F-X, et al. Predicting drug–target interaction using positive-unlabeled learning. Neurocomputing. 2016;206:50-7.

73. Shi Y, Zhang X, Liao X, Lin G, Schuurmans D. Protein-chemical interaction prediction via kernelized sparse learning SVM. Pac Symp Biocomput. 2013:41-52.

74. Peng L, Liao B, Zhu W, Li K. Predicting Drug-Target Interactions with Multi-information Fusion. IEEE J Biomed Health Inform. 2015.

75. Wang YC, Deng N, Chen S, Wang Y. Computational study of drugs by integrating omics data with kernel methods. Mol Inform. 2013;32(11-12):930-41.

76. Niu YQ. Supervised prediction of drug-target interactions by ensemble learning. J Chem Pharm Res. 2014;6(7):1991-9.

77. van Laarhoven T, Nabuurs SB, Marchiori E. Gaussian interaction profile kernels for predicting drug-target interaction. Bioinformatics. 2011;27(21):3036-43.

78. Hao M, Wang Y, Bryant SH. Improved prediction of drug-target interactions using regularized least squares integrating with kernel fusion technique. Anal Chim Acta. 2016;909:41-50.

79. Nascimento AC, Prudencio RB, Costa IG. A multiple kernel learning algorithm for drug-target interaction prediction. BMC Bioinformatics. 2016;17:46.

80. Liu Y, Wu M, Miao C, Zhao P, Li XL. Neighborhood regularized logistic matrix factorization for drug-target interaction prediction. PLoS Comput Biol. 2016;12(2):e1004760.

81. Mousavian Z, Khakabimamaghani S, Kavousi K, Masoudi-Nejad A. Drug-target interaction prediction from PSSM based evolutionary information. J Pharmacol Toxicol Methods. 2016;78:42-51.

82. Cao DS, Zhang LX, Tan GS, Xiang Z, Zeng WB, Xu QS, et al. Computational Prediction of Drug-Target Interactions Using Chemical, Biological, and Network Features. Mol Inform. 2014;33(10):669-81.

83. Yan XY, Zhang SW, Zhang SY. Prediction of drug-target interaction by label propagation with mutual interaction information derived from heterogeneous network. Mol BioSyst. 2016;12(2):520-31.

84. Koohi A, editor. Prediction of drug-target interactions using popular Collaborative Filtering methods. 2013 IEEE International Workshop on Genomic Signal Processing and Statistics; 2013: IEEE.

85. Zheng X, Ding H, Mamitsuka H, Zhu S, editors. Collaborative matrix factorization with multiple similarities for predicting drug-target interactions. Proceedings of the 19th ACM SIGKDD international conference on Knowledge discovery and data mining; 2013: ACM.

86. Ezzat A, Zhao P, Wu M, Li X, Kwoh CK. Drug-Target Interaction Prediction with Graph Regularized Matrix Factorization. 2016.

87. Yamanishi Y, Araki M, Gutteridge A, Honda W, Kanehisa M. Prediction of drug-target interaction networks from the integration of chemical and genomic spaces. Bioinformatics. 2008;24(13):i232-i40.

88. Gfeller D, Grosdidier A, Wirth M, Daina A, Michielin O, Zoete V. SwissTargetPrediction: a web server for target prediction of bioactive small molecules. Nucleic Acids Res. 2014;42(Web Server issue):W32-8.

89. Yamanishi Y, Kotera M, Moriya Y, Sawada R, Kanehisa M, Goto S. DINIES: drug-target interaction network inference engine based on supervised analysis. Nucleic Acids Res. 2014;42(Web Server issue):W39-W45.

90. Wang L, Ma C, Wipf P, Liu H, Su W, Xie XQ. TargetHunter: an in silico target identification tool for predicting therapeutic potential of small organic molecules based on chemogenomic database. AAPS J. 2013;15(2):395-406.

91. Keiser MJ, Roth BL, Armbruster BN, Ernsberger P, Irwin JJ, Shoichet BK. Relating protein pharmacology by ligand chemistry. Nat Biotechnol. 2007;25(2):197-206.

92. Liu X, Vogt I, Haque T, Campillos M. HitPick: a web server for hit identification and target prediction of chemical screenings. Bioinformatics. 2013;29(15):1910-2.

93. Reker D, Rodrigues T, Schneider P, Schneider G. Identifying the macromolecular targets of de novo-designed chemical entities through self-organizing map consensus. Proc Natl Acad Sci U S A. 2014;111(11):4067-72.

94. Yao ZJ, Dong J, Che YJ, Zhu MF, Wen M, Wang NN, et al. TargetNet: a web service for predicting potential drug-target interaction profiling via multi-target SAR models. J Comput Aided Mol Des. 2016;30(5):413-24.

95. He Z, Zhang J, Shi XH, Hu LL, Kong X, Cai YD, et al. Predicting drug-target interaction networks based on functional groups and biological features. PLoS One. 2010;5(3):e9603.

96. Hattori M, Tanaka N, Kanehisa M, Goto S. SIMCOMP/SUBCOMP: chemical structure search servers for network analyses. Nucleic Acids Res. 2010;38(Web Server issue):W652-6.

97. Mauri A, Consonni V, Pavan M, Todeschini R. Dragon software: an easy approach to molecular descriptor calculations. Match. 2006;56(2):237-48.

98. Yap CW. PaDEL-descriptor: An open source software to calculate molecular descriptors and fingerprints. J Comput Chem. 2011;32(7):1466-74.

99. Marrero-Ponce Y, Castillo-Garit JA, Olazabal E, Serrano HS, Morales A, Castanedo N, et al. TOMOCOMD-CARDD, a novel approach for computer-aided 'rational' drug design: I. Theoretical and experimental assessment of a promising method for computational screening and in silico design of new anthelmintic compounds. J Comput Aided Mol Des. 2004;18(10):615-34.

100. Tetko IV, Gasteiger J, Todeschini R, Mauri A, Livingstone D, Ertl P, et al. Virtual computational chemistry laboratory--design and description. J Comput Aided Mol Des. 2005;19(6):453-63.

101. Hong H, Xie Q, Ge W, Qian F, Fang H, Shi L, et al. Mold^2^, molecular descriptors from 2D structures for chemoinformatics and toxicoinformatics. J Chem Inf Model. 2008;48(7):1337-44.

102. Steinbeck C, Hoppe C, Kuhn S, Floris M, Guha R, Willighagen EL. Recent developments of the chemistry development kit (CDK) - an open-source java library for chemo- and bioinformatics. Curr Pharm Des. 2006;12(17):2111-20.

103. O'Boyle NM, Banck M, James CA, Morley C, Vandermeersch T, Hutchison GR. Open Babel: An open chemical toolbox. J Cheminform. 2011;3:33.

104. Rao HB, Zhu F, Yang GB, Li ZR, Chen YZ. Update of PROFEAT: a web server for computing structural and physicochemical features of proteins and peptides from amino acid sequence. Nucleic Acids Res. 2011;39(Web Server issue):W385-90.

105. Lagorce D, Sperandio O, Baell JB, Miteva MA, Villoutreix BO. FAF-Drugs3: a web server for compound property calculation and chemical library design. Nucleic Acids Res. 2015;43(W1):W200-7.

106. Gramatica P, Chirico N, Papa E, Cassani S, Kovarich S. QSARINS: A new software for the development, analysis, and validation of QSAR MLR models. J Comput Chem. 2013;34(24):2121-32.
